# Excitatory nucleo-olivary pathway shapes cerebellar outputs for motor control

**DOI:** 10.1038/s41593-023-01387-4

**Published:** 2023-07-20

**Authors:** Xiaolu Wang, Zhiqiang Liu, Milen Angelov, Zhao Feng, Xiangning Li, Anan Li, Yan Yang, Hui Gong, Zhenyu Gao

**Affiliations:** 1 Department of Neuroscience, Erasmus MC, Rotterdam, the Netherlands; 2 Department of Neurosurgery, Xiangya Hospital, Central South University, Changsha, China; 3 Research Unit of Multimodal Cross Scale Neural Signal Detection and Imaging, Chinese Academy of Medical Sciences, HUST-Suzhou Institute for Brainsmatics, JITRI, Suzhou, China; 4 State Key Laboratory of Digital Medical Engineering, School of Biomedical Engineering, Hainan University, Haikou, China; 5 Britton Chance Center for Biomedical Photonics, Wuhan National Laboratory for Optoelectronics, Huazhong University of Science and Technology, Wuhan, China; 6 State Key Laboratory of Brain and Cognitive Sciences, Institute of Biophysics, Chinese Academy of Sciences, Beijing, China; 7 University of Chinese Academy of Sciences, Beijing, China; 8 Institute of Artificial Intelligence, Hefei Comprehensive National Science Center, Hefei, China

**Keywords:** Cerebellum, Neural circuits

## Abstract

The brain generates predictive motor commands to control the spatiotemporal precision of high-velocity movements. Yet, how the brain organizes automated internal feedback to coordinate the kinematics of such fast movements is unclear. Here we unveil a unique nucleo-olivary loop in the cerebellum and its involvement in coordinating high-velocity movements. Activating the excitatory nucleo-olivary pathway induces well-timed internal feedback complex spike signals in Purkinje cells to shape cerebellar outputs. Anatomical tracing reveals extensive axonal collaterals from the excitatory nucleo-olivary neurons to downstream motor regions, supporting integration of motor output and internal feedback signals within the cerebellum. This pathway directly drives saccades and head movements with a converging direction, while curtailing their amplitude and velocity via the powerful internal feedback mechanism. Our finding challenges the long-standing dogma that the cerebellum inhibits the inferior olivary pathway and provides a new circuit mechanism for the cerebellar control of high-velocity movements.

## Abstract (web summary)

Complex spikes (CSs) driven by inferior olivary neurons have crucial roles in motor control. Wang et al. identified an excitatory pathway from the cerebellar nuclei to the inferior olive that drives rapid feedback CSs and contributes to the fine control of ocular and body movements.

## Main

It is a central function of the brain to generate predictions of future action during ongoing movements to achieve optimal spatiotemporal precision^1–4^. The mismatch between the predictive motor commands and the sensory feedback signals generates prediction errors. A large body of literature has provided supporting evidence for the vital roles of predictive error in updating ongoing motor command, canceling sensory corollary discharge and instructing future movements^1,2^. The cerebellum is a key region that generates predictive motor commands by constructing and maintaining the internal forward models. The cerebellar computation is supported by its highly organized nucleo-olivo-cortical modules^5,6^. Inferior olivary (IO) neurons integrate the extracerebellar feedback excitation and the cerebellar inhibition^7,8^ to generate complex spike (CS) discharges in Purkinje cells (PCs). Mounting evidence has illustrated the functional importance of CSs in signaling predictive errors and directing movements^9–11^. For instance, during saccadic eye movements, simple spikes (SSs) encode real-time motion in the PC clusters that share common climbing fiber inputs^10^. These data indicate that the tuning preference of CSs frames the kinetics of movements.

Yet, high-velocity movements, such as saccadic eye movements, stand out because they are too fast for sensory feedback-based online control. Indeed, one of the remaining enigmas in motor control theory is how animals could precisely steer eye movements independent of the visual and proprioceptive feedbacks^12,13^. The corollary discharge generated internally in the cerebellum might be most sufficient for online control of high-velocity movements^1,13^. Several recent studies have also suggested that CS activity could emerge internally from cerebellar computation and encode predictive signals for future movements^14–16^. During saccadic adaptation, CS gradually emerges as the saccades adapt to a new target position^17^. This is not in line with an error CS signal as the visual error gradually diminishes during adaptation. Interestingly, the CS modulation is most prominent after saccade onset and the modulation amplitudes encode saccade kinematics^18^. Therefore, it is reasonable to speculate that CS encodes an efference copy of the cerebellar motor command, which provides powerful feedback signals to the cerebellar cortex. The main challenge in reconciling such cerebellar-driven CSs is the long-standing doctrine that the cerebellar nucleus (CN), the main cerebellar output neurons, provides exclusively GABAergic projection to IO and suppresses CS activity during behavior^8,19^. Therefore, how cerebellar output drives internal feedback CSs within millisecond precision, independent of extracerebellar excitation, remains elusive.

Using comprehensive anatomical, electrophysiological and functional assays, we demonstrate that the medial cerebellum implements a unique excitatory nucleo-olivary circuitry to generate internal feedback CSs and modulate the kinematics of high-velocity movements in this study.

## Results

### Distinct excitatory and inhibitory FN–IO projections

**Fig. 1:**
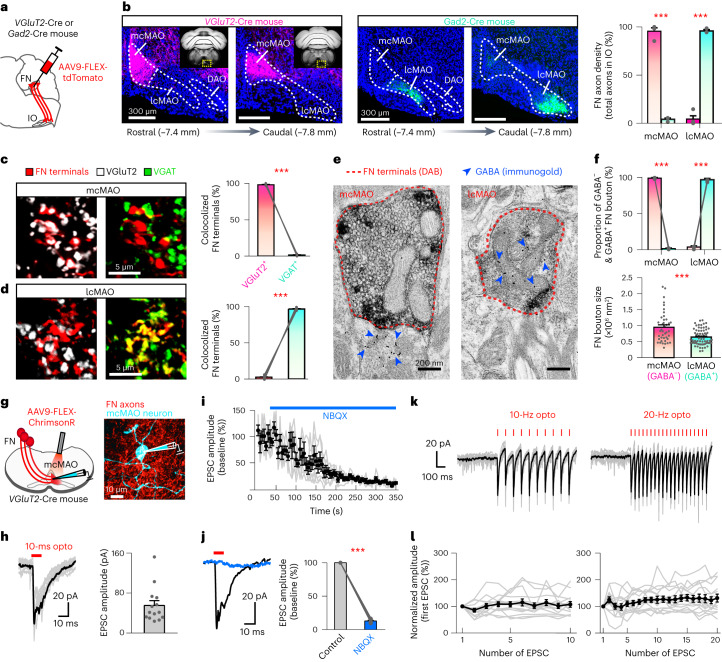
FN provides excitatory and inhibitory projections to distinct IO regions. **a**, Anterograde tracing for labeling the excitatory or inhibitory FN outputs. **b**, Distribution of the excitatory and inhibitory FN axons in cMAO (*n* = 4 and 3 for *VGluT2*-Cre and *Gad2*-Cre mice, respectively; two-sided *t*-test, mcMAO, *P* = 3.90 × 10^−6^; lcMAO, *P* = 3.90 × 10^−6^). Dots represent individual mice, and bars represent mean + s.e.m. **c**,**d**, Fraction of VGluT2^+^ and VGAT^+^ FN terminals in the mcMAO and lcMAO (two-sided paired *t*-test, *n* = 15 and 19 sections from three mice for **c** and **d**, respectively. *P* = 1.12 × 10^−25^ for **c** and *P* = 9.57 × 10^−28^ for **d**. Dots represent individual sections, and bars represent mean + s.e.m. **e**, Electron microscopy images of DAB-labeled FN terminals (dashed contours) in mcMAO (left) and lcMAO (right). Inhibitory terminals are identified by the presence of dense GABA-immunogold labeling (blue arrowheads). **f**, Upper: fraction of GABA^−^ and GABA^+^ FN boutons in mcMAO and lcMAO (two-sided paired *t*-test, *n* = 4 mice, *P* = 1.87 × 10^−5^ for mcMAO, *P* = 1.44 × 10^−4^ for lcMAO). Dots represent individual mice, and bars represent mean + s.e.m. Lower: comparison of the bouton sizes between GABA^−^ and GABA^+^ FN terminals (GABA^−^, *n* = 41 and GABA^+^, *n* = 68 from four mice; two-sided *t*-test, *P* = 2.11 × 10^−5^). Dots represent individual boutons, and bars represent mean + s.e.m. **g**, Left: whole-cell recording of mcMAO neurons while photoactivating FN axons. Right: example mcMAO neuron (biotin labeled, 1 of 14 cells displayed). **h**, Left: optogenetic evoked EPSCs from an example recoding (dark, average and gray, 10 individuals). Right: EPSC amplitudes of 14 cells from five mice. Dots represent individual cells, bars represent mean + s.e.m. **i**, Changes in EPSC amplitudes over time following NBQX infusion (*n* = 6 cells). Dots and bars represent mean ± s.e.m. **j**, Example EPSC traces and summary of their amplitudes before and after NBQX application (*n* = 6 cells from four mice; two-sided paired *t*-test, *P* = 4.92 × 10^−9^). Dots represent individual cells, and bars represent mean + s.e.m. **k**, EPSCs following 10- and 20-Hz train photoactivation. **l**, EPSC amplitudes following each light pulse (normalized to the first EPSC, *n* = 14 cells from four mice). Dots and bars represent mean ± s.e.m. Source data

**Extended Data Fig. 1:**
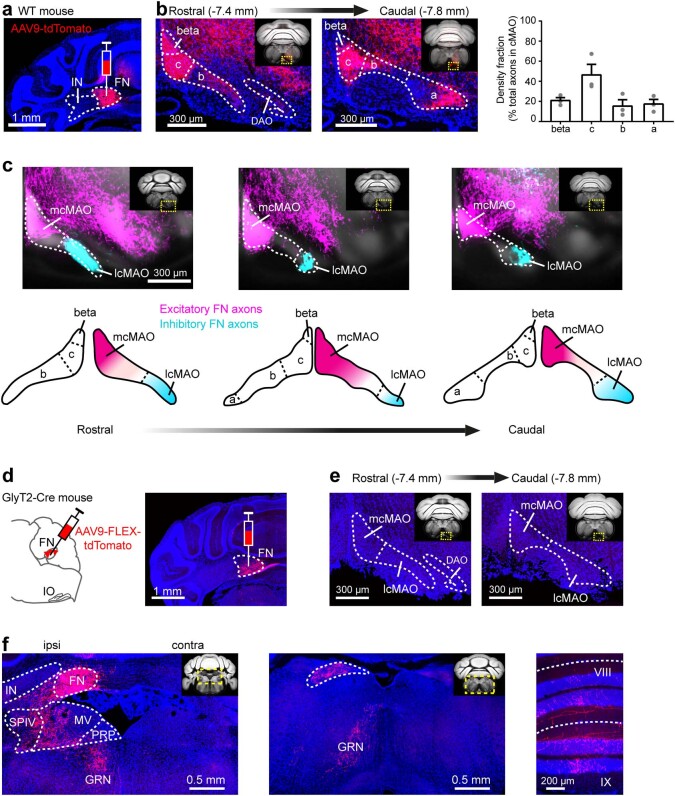
Distribution of FN axons in the cMAO. Related to (Fig. 1a–d). **a**, Bulk injection of anterograde AAV9-tdTomato unilaterally in the FN (1/3 mice displayed). **b**, Representative images along the rostral-caudal axis, and quantification of FN axons in four subnuclei of the cMAO (*n* = 3 mice, mean ± s.e.m.). The projection density in each subnucleus is normalized to the total axonal labeling in the cMAO. **c**, Summary of the excitatory and inhibitory FN projections in the cMAO (related to the experiments in Fig. 1a,b). Top: superimposed cMAO images from VGluT2-Cre (magenta, 1/4 mice displayed) and Gad2-Cre (cyan, 1/3 mice displayed) mice injected with AAV9-FLEX-tdTomato in the FN. Raw images are registered in the Allen Mouse Brain CCF. Bottom: graphic illustration of excitatory and inhibitory FN projections in the cMAO. The region that receives excitatory FN inputs mainly consists of beta, c, and the medial part of b subnuclei, which is termed the medial cMAO (mcMAO_,_ magenta region). The region that receives inhibitory FN inputs consists of a and the lateral part of b subnuclei, which is termed the lateral cMAO (lcMAO_,_ cyan regions). cMAO: caudal medial accessory olive, FN: fastigial nucleus, IN: interposed nucleus, DAO: dorsal accessory olive. **d**, Tracing strategy (left) and representative image (right) showing AAV9-FLEX-tdTomato injected into the FN of a GlyT2-Cre mouse. Glycinergic FN neurons were selectively labeled by this approach. **e**, Histological sections of cMAO regions showing the absence of glycinergic FN axons. **f**, Glycinergic FN axons were observed in the vestibular and reticular nuclei regions of the medulla (left and middle images), as well as in the cerebellar vermis (as the nucleo-cortical mossy fibers, right image) ipsilateral to the injection site. Experiments were repeated in 3 animals. DAO: dorsal accessory olive, FN: fastigial nucleus, GRN: gigantocellular reticular nucleus, IN: interposed nucleus, LV: lateral vestibular nucleus, MV: medial vestibular nucleus, PRP: prepositus nucleus, SPIV: spinal vestibular nucleus. Source data

The medial CN (fastigial nucleus, FN) sends dense projections to confined regions in the caudal medial accessory olive^20^ (cMAO; Extended Data Fig. 1a,b). To characterize the synaptic properties of FN-cMAO projections, we selectively labeled the excitatory and inhibitory FN neurons by injecting AAV9-FLEX-tdTomato into the FN of *Slc17ac*-Cre (*VGluT2*-Cre) and *Gad2*-Cre mice, respectively (Fig. 1a). Dense axons were observed in the medial part of cMAO (termed mcMAO) of the *VGluT2*-Cre mice, and GABAergic FN axons were observed in the lateral part of cMAO (termed lcMAO; Fig. 1b) of the *Gad2*-Cre mice. Immunohistological staining for the glutamatergic marker VGluT2 and the GABAergic marker VGAT confirmed that FN terminals in the mcMAO region were predominantly glutamatergic, whereas those in the lcMAO region were predominantly GABAergic (Fig. 1c,d). The minimal overlap between the glutamatergic and GABAergic FN projections in the IO (Extended Data Fig. 1c) suggests that these two pathways are geometrically segregated. We further verified the nature of neurotransmitters in the FN–IO terminals using immunoelectron microscopy. Postembedding immunogold-labeled GABA was enriched in the FN synaptic terminals in the lcMAO region but largely absent in the FN terminals in the mcMAO region (Fig. 1e,f). The GABA^−^ FN-mcMAO synapses contain densely packed round synaptic vesicles, a unique feature of excitatory synapses. In addition, the GABA^−^ (excitatory) FN-mcMAO synapses are substantially larger than the GABA^+^ (inhibitory) FN-lcMAO synapses (Fig. 1f).

**Extended Data Fig. 2:**
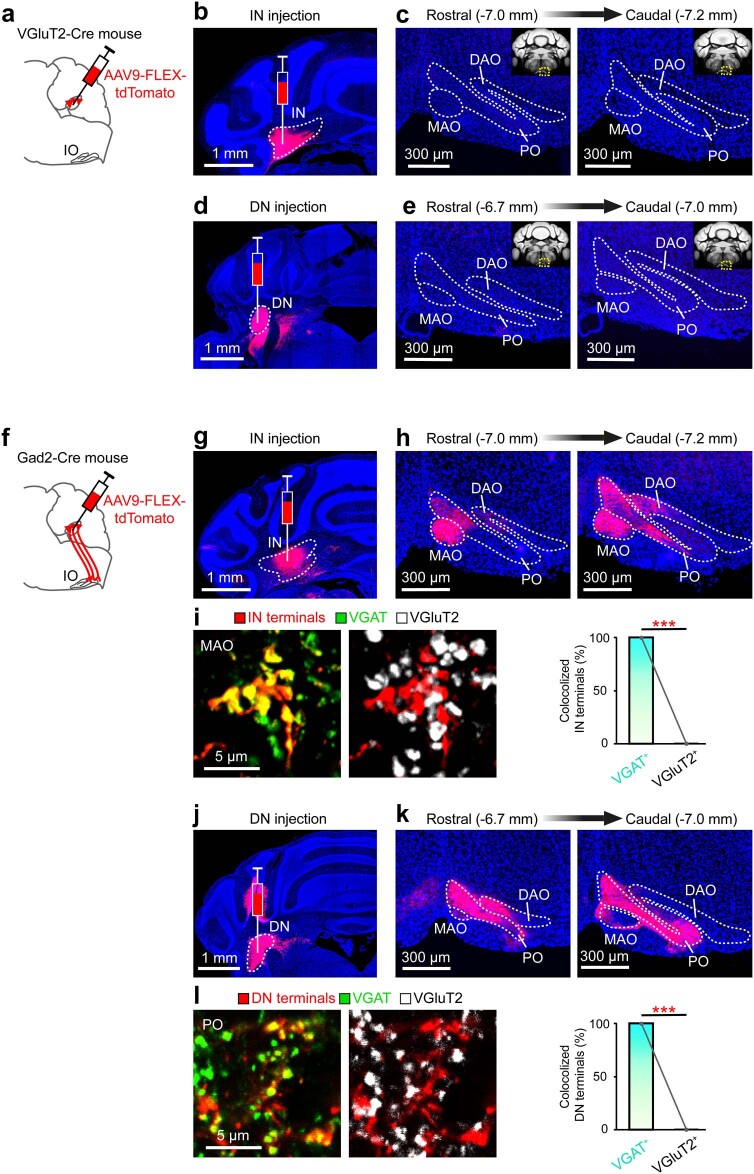
IN and DN provide exclusive inhibitory projections into distinct IO regions. **a**, Injection strategy for labeling the excitatory projections from the IN and DN (*n* = 3 mice for each nucleus). **b**, **c**, Representative sections showing the injection site in the IN and the absence of VGluT2-positive axons in the IO. **d**, **e**, Same as (**b**, **c**), but for the injection in the DN. **f**, Same as (**a**), but for labeling the inhibitory projections from the IN and DN (*n* = 3 mice for each group). **g**, **h**, Example sections showing the injection site in the IN and dense GABAergic axonal labeling in the contralateral MAO and DAO. **i**, Confocal images of IN terminals in the MAO region, showing strong colocalization with the GABAergic synaptic marker VGAT, but not with the glutamatergic synaptic marker VGluT2 (*n* = 8 IO sections, two-sided paired *t*-test, *P* = 1.53×10^−16^). **j**-**l**, Same as (**g**-**i**), but for injections in the DN of Gad2-Cre mice. Dense projections from the DN were found mainly in the contralateral PO, colocalizing with VGAT staining, but not with VGluT2 staining (*n* = 11 IO sections, two-sided paired *t*-test, *P* = 1.03×10^−22^). DAO: dorsal accessory olive, DN: dentate nucleus, IN: interposed nucleus, PO: principal olive, MAO: medial accessary olive. Source data

Colocalization of glycine and GABA has been reported in FN neurons^21^. To examine whether the glycinergic FN neurons project to the IO, we injected AAV9-FLEX-tdTomato into the FN of *GlyT2*-Cre mice (Extended Data Fig. 1d). In contrast to the discrete glutamatergic and GABAergic FN–IO projections, glycinergic FN axons were not found in the IO (Extended Data Fig. 1e,f). Therefore, the inhibitory FN-lcMAO projection is exclusively GABAergic. Interestingly, glutamatergic CN–IO projections were not observed from the interposed nucleus (IN) or dentate nucleus (DN; Extended Data Fig. 2a–e). The IN and DN projections in the IO were exclusively GABAergic (Extended Data Fig. 2f–l), consistent with previous studies^7^. Collectively, our results reveal a previously unknown glutamatergic CN–IO pathway in the medial cerebellum that is distinct from the intermediate and lateral cerebello-olivary modules.

To assess the electrophysiological properties of the glutamatergic FN-mcMAO synapses, we expressed ChrimsonR specifically in the glutamatergic FN neurons and performed in vitro patch-clamp recordings of mcMAO neurons (Fig. 1g). Transient optogenetic activation of the ChrimsonR-expressing FN axons triggered excitatory postsynaptic currents (EPSCs; amplitude = 55.3 ± 9.5 pA) in mcMAO neurons (Fig. 1h). Application of the AMPA receptor antagonist NBQX diminished the photo-evoked EPSC amplitudes by 87% (Fig. 1i,j), indicating that FN-mcMAO synaptic transmission is predominantly mediated by AMPA receptors. Train photoactivation of FN axons at 10- and 20-Hz induced sustained synaptic transmission without noticeable decreases in EPSC amplitudes (Fig. 1k,l). These results indicate that the FN-mcMAO synapses support both transient and tonic synaptic transmission. In contrast, optogenetic activation of the FN axons in the lcMAO triggered inhibitory postsynaptic currents (IPSCs; amplitude = 76.4 ± 10.4 pA) in the lcMAO neurons (Supplementary Fig. 1). The IPSCs were abolished by application of GABA_A_ receptor antagonist picrotoxin, confirming GABAergic transmission at the FN-lcMAO synapses. These data demonstrate that the medial cerebellum diverges its excitatory and inhibitory outputs into distinct IO regions.

### Input–output organizations of the nucleo-olivary pathways

**Extended Data Fig. 3:**
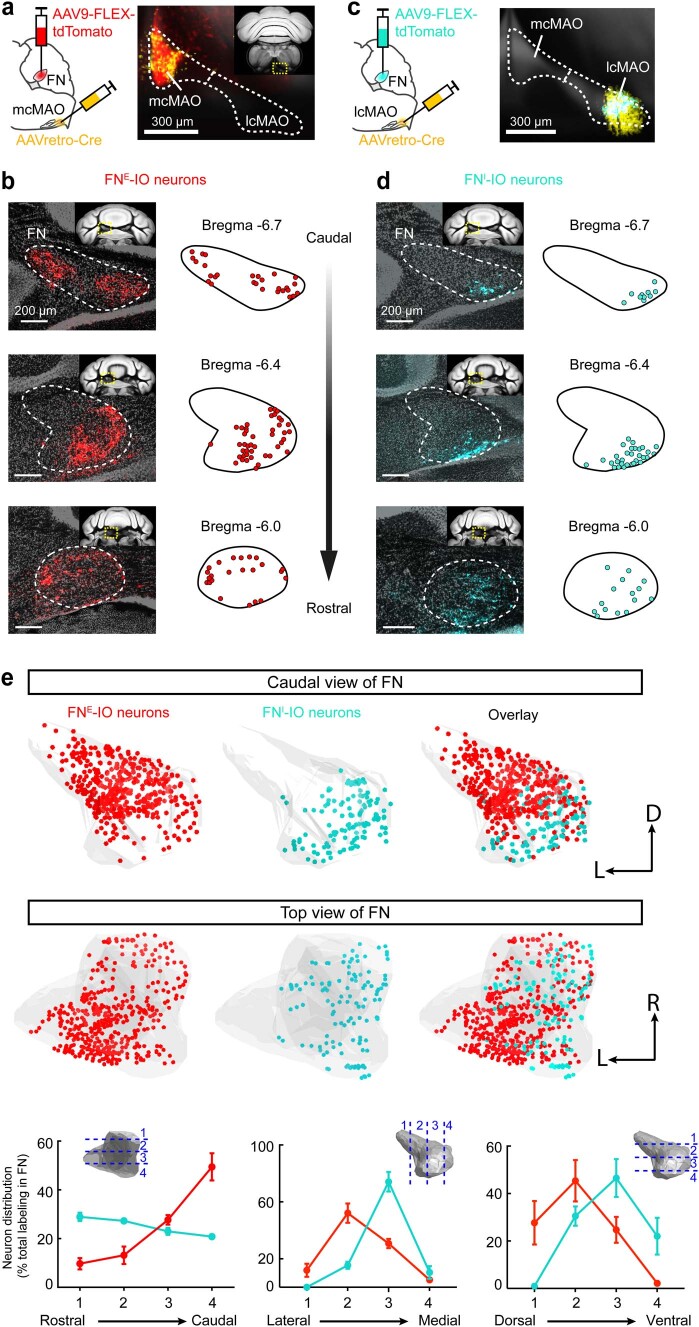
Distribution of the FN^E^-IO and FN^I^-IO neurons in the FN. **a**, Tracing strategy for labeling excitatory mcMAO-projecting FN neurons (termed FN^E^-IO neurons). AAV9-FLEX-tdTomato was injected into the FN, and AAVretro-Cre was injected into the mcMAO. CTB-Alexa647 was co-injected with AAVretro-Cre to label the injection site, showing colocalization of FN axons (red) and Alexa647 (yellow) in the mcMAO. **b**, Histological sections, and the corresponding annotation of FN^E^-IO neurons from a representative animal. **c**, **d**, Same as (**a**, **b**), but for labeling the inhibitory lcMAO-projecting FN neurons (termed FN^I^-IO neurons). AAV9-FLEX-tdTomato was injected into the FN, and AAVretro-Cre (mixed with CTB-Alexa647) was injected into the lcMAO. Example image showing colocalization of FN axons (turquoise) and Alexa647 (yellow) in the lcMAO region. **e**, Distribution of FN^E^-IO (red) and FN^I^-IO neurons (turquoise, *n* = 3 mice for each group, mean ± s.e.m.). Upper: caudal and top views of three-dimensionally rendered FN and the superimposed FN^E^-IO and FN^I^-IO neurons. Lower: quantifications of these two populations along the rostro-caudal, latero-medial, and dorso-ventral axes. Stereotaxic abbreviations, D: dorsal, L: lateral, R: rostral. Source data

FN neurons can be classified into several subgroups that present distinct biomarker immunoreactivity, as well as segregate anatomical localization^20^. To understand the anatomical distribution of the mcMAO-projecting excitatory FN neurons (FN^E^–IO) and lcMAO-projecting inhibitory FN neurons (FN^I^–IO), we registered these two populations to the Allen Mouse Brain Common Coordinate Framework (CCF; Methods) and compared their distributions in the FN. The FN^E^–IO neurons were widely distributed in the FN but were most dense in the caudal FN, including the dorsolateral protuberance. In contrast, the FN^I^–IO neurons formed a thin sheet at the ventral FN and were distributed evenly along the anterior–posterior axis (Extended Data Fig. 3).

**Fig. 2:**
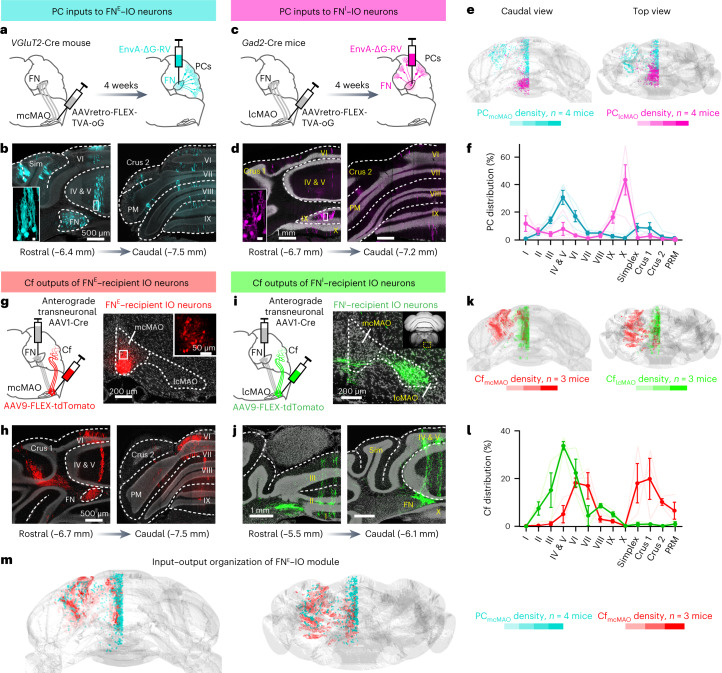
Distinct input–output organizations of the excitatory FN^E^–IO and inhibitory FN^I^–IO modules. **a**, Experimental schematic of cell-type-specific retrograde transneuronal tracing of PC inputs to FN^E^–IO neurons. **b**, Example images of retrogradely labeled PCs in the cerebellar cortex (1 of 4 mice displayed). Inset scale bar = 10 µm. **c**,**d**, Same as **a** and **b**, but for monosynaptic rabies tracing of the PC inputs to FN^I^–IO neurons (1 of 4 mice displayed). **e**,**f**, Comparison of the distribution of PC inputs to FN^E^–IO and FN^I^–IO neurons. **e**, Three-dimensional rendering of all labeled PC projections to FN^E^–IO neurons (turquoise, *n* = 4 mice) and FN^I^–IO neurons (magenta, *n* = 4 mice). **f**, Distribution of labeled PCs across cerebellar lobules. Density is normalized to the total labeled PCs of each animal. Lighter curves represent individual mice, darker dots and bars represent mean ± s.e.m. **g**, Experimental schematic of tracing the climbing fibers originating from FN^E^-recipient neurons in the mcMAO. **h**, Example images of labeled climbing fibers in the cerebellar cortex (1 of 3 mice displayed). **i**,**j**, Same as **g** and **h**, but for tracing the climbing fibers from FN^I^-recipient IO neurons (1 of 3 mice displayed). **k**,**l**, Same as **e** and **f**, but for comparison of climbing fiber distribution from FN^E^-recipient IO neurons (red, *n* = 3 mice) and FN^I^-recipient IO neurons (green, *n* = 3 mice). Lighter curves represent individual mice, darker dots and bars represent mean ± s.e.m. **m**, Overlay of PCs (inputs) and climbing fibers (outputs) reveals discrete excitatory nucleo-olivo-cortical modules for the FN^E^–IO pathway. Source data

PCs in the vermal and paravermal regions project to the FN and receive climbing fiber inputs from the cMAO^22^. The compartmentalized distribution of excitatory and inhibitory IO-projecting neurons in the FN suggests that these groups of FN neurons might be innervated by specific cerebellar cortical regions, thereby forming discrete nucleo-olivo-cortical modules. To test this hypothesis, we first used cell-type-specific transneuronal rabies virus to identify the PC input patterns of the FN^E^–IO module. We injected AAVretro-FLEX-TVA-oG into the mcMAO of *VGluT2*-Cre mice (Fig. 2a), permitting the expression of avian tumor virus receptor A (TVA) and rabies glycoprotein (oG) in the FN^E^–IO neurons that facilitate monosynaptic rabies virus retrograde tracing^23^. Subsequent injection of EnvA-pseudotyped G-deleted rabies virus in the FN selectively transfected the TVA-oG-expressing FN^E^–IO neurons and thereby retrogradely labeled the PCs that project to these starter FN neurons. We observed rabies-labeled PCs in several cerebellar cortical areas, specifically concentrated in two parasagittal regions, including the region of vermal lobules III to VI and the region of paravermal simplex lobule and crus 1 (Fig. 2b,f). We next applied a similar strategy to identify the PC inputs to the FN^I^–IO module (Fig. 2c). This group of PCs was found predominantly in posterior vermal lobules IX and X (Fig. 2d,f). Superimposing the two PC populations that project to the FN^E^–IO and FN^I^–IO neurons revealed largely segregated compartments in the cerebellar cortex (Fig. 2e,f), consistent with the functional module hypothesis of the cortico-nucleo-olivary circuits^20,22^.

The organized olivo-cerebellar zonation suggests that different IO compartments send climbing fibers to distinct regions in the cerebellar cortex^24^. We next examined the distribution of climbing fibers originating specifically from the FN-recipient neurons in the mcMAO by injecting anterograde transneuronal AAV1-Cre into the FN and AAV9-FLEX-tdTomato into the mcMAO (Fig. 2g). AAV1-Cre enabled anterograde transneuronal infection of postsynaptic neurons^25^, thereby selectively labeling FN-recipient neurons in the mcMAO and their climbing fibers in the cerebellar cortex (Fig. 2g). Climbing fibers were found specifically in several cortical regions, but most concentrated in the vermal lobules IV to VII, and the paravermal simplex lobule and the crus 1 and 2 (Fig. 2h,i). In contrast, climbing fibers originating from the FN-recipient neurons in the lcMAO were mostly concentrated in the anterior vermal lobules but not the paravermal region (Fig. 2i–l), consistent with a previous study on olivo-cerebellar projections^26^. The extensively overlapping patterns between PC inputs and the climbing fiber output for the excitatory FN^E^–IO pathway (Fig. 2m) suggest that this nucleo-olivo-cortical module could form closed-loops and provide internal feedback signals to specific cerebellar modules. In summary, our transneuronal retrograde and anterograde tracing results illustrate the relationship between the PC input and climbing fiber output for the excitatory FN^E^–IO and inhibitory FN^I^–IO pathways and highlight different functional modules within the cortical–FN–IO circuits.

### FN^E^–IO pathway drives CSs to shape cerebellar outputs

**Fig. 3:**
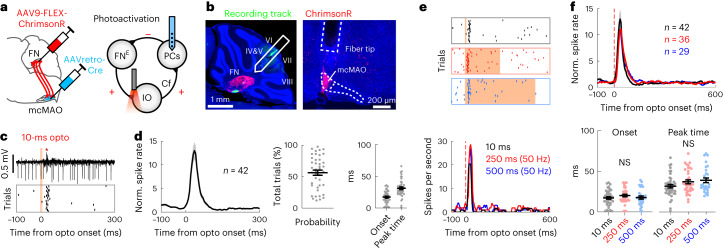
Excitatory nucleo-olivary pathway drives feedback CSs. **a**, Schematics illustrating viral injection and PC recording while photoactivating FN axons in the mcMAO. Excitatory and inhibitory projections are denoted as + and −, respectively. **b**, Histological images showing left, ChrimsonR (red) expression in the FN neurons and DiO-labeled recording track (green) in the posterior vermis (1 of 6 mice displayed) and right, optic fiber tip over the mcMAO. **c**, Example PC recording (asterisk represents CS) and raster plot of CS activity following 10-ms photoactivation. **d**, Average PSTH (mean ± s.e.m.), probability, onset and peak timing of photoactivated CSs (*n* = 42 PCs from six mice). Dots represent individual neurons, and bars represent mean ± s.e.m. **e**, Raster plots and PSTH traces of CS activity from an example vermal PC, following 10-ms single pulse (black), 250-ms train (red, 50 Hz and 50% duty cycle) and 500-ms train (blue, 50 Hz and 50% duty cycle) stimulations. The photoactivation intensity was 3.0 mW for all protocols. **f**, Summary of CS activities from all PCs. Upper: average PSTHs (mean ± s.e.m.). The numbers of cells for individual conditions are shown in the panel, from six mice. Lower: timing of CS modulation following different photoactivation protocols (one-way ANOVA with Tukey’s multiple comparisons, *P* = 0.37 for onset and *P* = 0.09 for peak time). Dots represent individual neurons, and bars represent mean ± s.e.m. Source data

Activation of inhibitory CN neurons suppresses CS activity^27^, but the impact of the FN^E^–IO pathway on IO neurons and CS generation is unknown. We expressed ChrimsonR selectively in the FN^E^–IO neurons and recorded PC activity while photoactivating FN axons in the mcMAO in awake mice (Fig. 3a,b). We focused primarily on the vermal lobules V–VIII, in which the PCs are anatomically connected to the excitatory FN^E^–IO pathway (Fig. 3b and see also Fig. 2). A brief optogenetic activation (10-ms single pulse) robustly evoked CSs (Fig. 3c) with an average trial-by-trial evoked probability of 56.4 ± 4.1%, a modulation onset timing of 17.2 ± 1.5 ms and a peak timing of 30.9 ± 2.0 ms (Fig. 3d). Prolonged photoactivation of the FN terminals in mcMAO triggered similar CS activity patterns in the vermal PCs (Fig. 3e,f), suggesting that sustained FN excitatory inputs drive time-locked CSs after the photoactivation onset. Repetitive CSs were induced following each stimulus of a 10-Hz train photoactivation, yet the decreased probabilities indicate that FN^E^–IO activation evoked CSs in constrained frequencies (Supplementary Fig. 2). These results uncover an internal cerebellar mechanism to drive CS activities in PCs.

**Fig. 4:**
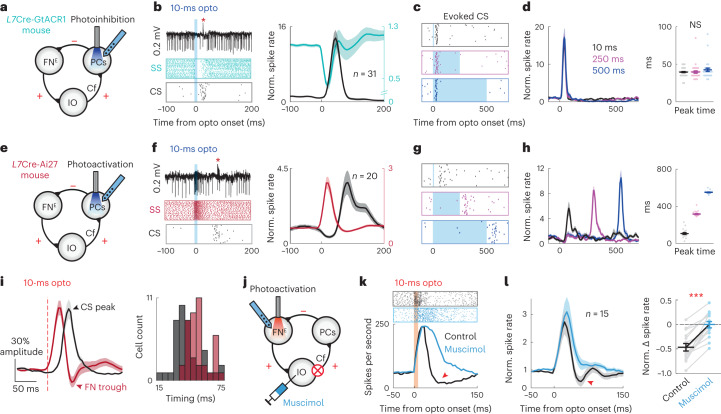
Vermal PCs bidirectionally modulate CS firing via the FN^E^–IO pathway and shape medial cerebellar outputs. **a**, Schematic illustrating vermal PC recording while photoinhibiting PCs in the same region. **b**, Left: example PC recording and raster plot of SSs and CSs following 10-ms photoinhibition. Right: population summary of the SS (turquoise) and CS (black) responses (*n* = 31 PCs from 4 mice). Activity traces are plotted as mean ± s.e.m. **c**, Example raster plots of CS activity following 10-ms, 250-ms and 500-ms PC photoinhibition. **d**, Population summary of the spike rates and peak timings of evoked CSs (*n* = 31, 37 and 32 PCs for each condition from 4 mice). Left: PSTH traces are plotted as mean ± s.e.m.; right, dots represent individual cells and bars represent mean ± s.e.m. (one-way ANOVA with Tukey’s multiple comparisons, *P* = 0.32). **e**–**h**, Same as (**a**–**d**), but for vermal PC recording while photoactivating PCs in the same region. Color codes in (**g**) and (**h**) indicate different photoactivation durations as those in (**c**) and (**d**), *n* = 20, 25 and 21 PCs for each condition from 3 mice. **i**, Left: average PSTHs (mean ± s.e.m.) of FN neuron activity (*n* = 40) and CS activity (*n* = 42 PCs) following 10-ms photoactivation of the FN^E^–IO neurons. See experimental strategies for FN recording in Extended Data Fig. 6, and for PC recording in Fig. 3. Right: distribution histogram of CS peak timing (black bars) and FN trough timing (red bars). **j**, Schematic showing recording and activation of FN^E^–IO neurons before and after pharmacologically inhibiting mcMAO. **k**, Raster plots and PSTHs of an example FN neuron before (black) and after (blue) muscimol infusion in the mcMAO. Red arrowhead indicates the putative CS-mediated feedback inhibition, which is abolished after mcMAO inhibition. **l**, Group average FN neuron activity (mean ± s.e.m.) and comparison of the putative CS-mediated feedback inhibition before and after muscimol infusion (*n* = 15 from 6 mice, two-sided paired *t*-test, *P* = 1.47 × 10^−6^). Dots represent individual cells and bars represent mean ± s.e.m. Source data

**Extended Data Fig. 4:**
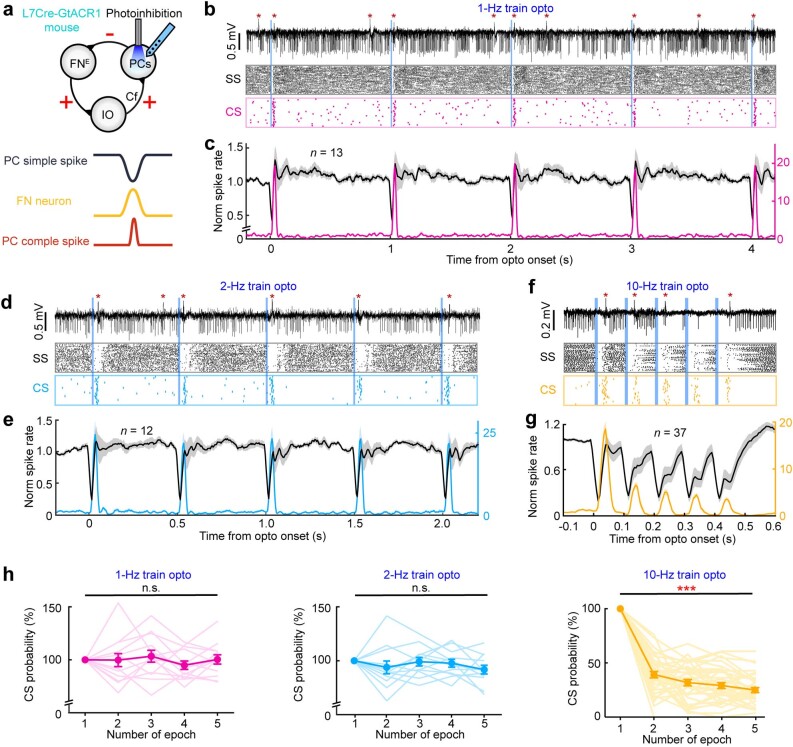
Inhibiting SS activity of vermal PCs entrains feedback CS activity via the excitatory FN^E^-IO module. Related to (Fig. 4a-d). **a**, Top: Experimental schematic, same as (Fig. 4a). Bottom: hypothetical effects of SS suppression on the disinhibition of FN neurons and subsequent activation of CSs in the same PC. **b**, Raw recording and raster plots of SS and CS activities from an example vermal PC, following 1-Hz PC inhibition (10-ms photoinhibition, 5 pulses per trial). **c**, Average PSTHs of SS (black) and CS (magenta) activities following 1-Hz PC inhibition (*n* = 13 from 3 mice, mean ± s.e.m.), showing CS activation following SS suppression. **d**, **e**, Same as (**b**, **c**), but for SS and CS activities following 2-Hz PC inhibition (10-ms photoinhibition, 5 pulses per trial; *n* = 12 from 3 mice, mean ± s.e.m.). **f**, **g**, Same as (**b**, **c**), but for SS and CS activities following 10-Hz PC inhibition (10-ms photoinhibition, 5 pulses per trial; *n* = 37 from 4 mice, mean ± s.e.m.). The optogenetic light intensity was 3.0 mW for all protocols. **h**, CS probabilities following three different train stimulations of SSs. The evoked probability is normalized to the first response. One-way ANOVA with Tukey’s multiple comparisons, left to right: *P* = 0.62, 0.33, and 5.8×10^−36^; *n* = 13 neurons from 3 mice, 12 neurons from 3 mice, and 37 neurons from 4 mice. Lighter curves represent individual neurons, darker dots and bars represent mean ± s.e.m. Source data

PCs can bidirectionally modulate their SS activity, resulting in upregulation/downregulation of CN outputs^28,29^. We next investigated the impacts of SS modulations on the feedback CS activity via the FN^E^–IO–cortical loop. Transient optogenetic inhibition of the vermal PCs in *L7*Cre-GtACR1 mice^30^ (Fig. 4a) resulted in a rapid SS suppression followed by an increase in CS firing rates (peak time 39.5 ± 1.1 ms) in the same PCs (Fig. 4b). The 250- and 500-ms PC inhibitions induced similar CS activity patterns that were time-locked to the photoinhibition onsets (Fig. 4c,d), despite the prolonged SS suppression (Supplementary Fig. 3). To rule out the possibility that CS activity following vermal PC inhibition is a movement-related feedback signal, we recorded PC activities in anesthetized animals (Supplementary Fig. 4a) in which their movements were completely abolished. Similar to the results from awake animals, 250-ms PC inhibition evoked well-timed CS activation (Supplementary Fig. 4b), further supporting the FN^E^–IO–cortical loop. These effects were consistent with the results induced by photoactivation of FN axons in mcMAO, suggesting that suppressing PC activity disinhibits the downstream FN^E^–IO neurons, and thereby drives feedback CSs in the PCs within the same module. To further examine the temporal constraints of this CS activation, we applied repetitive transient inhibition of PCs at different frequencies. Both 1- and 2-Hz train stimuli induced highly reliable CS activation, whereas the 10-Hz train stimulus resulted in CS firing with decreased evoke probabilities following each consecutive stimulus (Extended Data Fig. 4).

Activating PCs inhibits the intrinsic activity of CN neurons and induces a subsequent postinhibitory rebound firing after the cessation of CN inhibition^31^. We next examined whether activating vermal PCs could induce postinhibitory rebound activation in FN^E^–IO neurons, and thereby drive feedback CSs (Supplementary Fig. 5a). Indeed, optogenetic activation of the vermal PCs in *L7*Cre-Ai27 mice^32^ resulted in spike rate suppression followed by rebound activation in FN neurons (Supplementary Fig. 5b). Recordings from the vermal PCs revealed an SS facilitation followed by an increased CS activity at an average of 107.3 ms after the stimulation onset (10-ms optogenetic stimulation; Fig. 4e,f). Prolonged PC activation postponed the evoked CS peak timing, which was nevertheless time-locked to the photoactivation offset (Fig. 4g,h). By comparing the timings of FN rebound and CS facilitation, we found that the photo-evoked CSs occurred shortly after the FN rebound peak (Supplementary Fig. 5c); therefore, the increased CS activity is likely to be driven by the rebound activation of the FN^E^–IO neurons. Together with the PC inhibition experiments, our data suggest that bidirectional modulation of SS activity in vermal PCs directly activates CSs in the same cerebellar module via the feedback FN^E^–IO–cortical loop.

**Extended Data Fig. 5:**
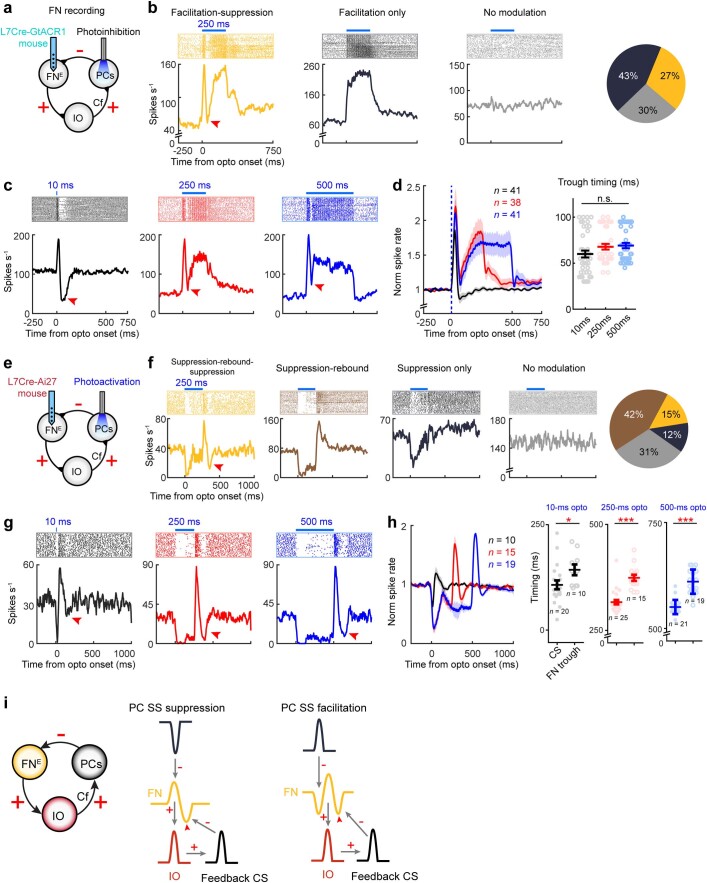
Bidirectional manipulations of vermal PCs induce post-faciliatory inhibition in a subpopulation of FN neurons. Related to (Fig. 4). **a**, Schematic of FN recording while photoinhibiting vermal PCs. **b**, Example FN neurons exhibiting different types of responses following a 250-ms PC train photoinhibition (50 Hz, 50% duty cycle). Yellow: a neuron exhibiting initial facilitation followed by transient inhibition (red arrowhead); black: a neuron exhibiting only facilitation; gray: an unresponsive neuron. Pie chart showing the proportion of each type among 111 FN neurons from 3 mice. **c**, **d**, From the FN subpopulation as in (**b**, yellow group), these neuron responses to PC photoinhibition by a 10-ms single pulse, 250-ms train (50 Hz, 50% duty cycle), and 500-ms train (50 Hz, 50% duty cycle), showing time-locked (to the photoinhibition onset) post-faciliatory inhibition pattern. See example neuron activities in (**c**) and summary in (**d**). **d**, Left: average PSTHs (mean ± s.e.m.) under each condition. Numbers of recordings are indicated in the panel, from 3 mice. Right: comparison of trough timing of each group (one-way ANOVA with Tukey’s multiple comparisons, *P* = 0.10). Circles represent individual neurons, bars represent mean ± s.e.m. **e**, Schematic of FN recording while photoactivating vermal PCs. **f**, Example FN neurons exhibiting different types of responses following 250-ms PC photoactivation. Yellow: a neuron exhibiting post-inhibitory rebound firing followed by subsequent suppression (red arrowhead); brown: a neuron exhibiting only post-inhibitory rebound facilitation; black: a neuron exhibiting only suppression; gray: an unresponsive neuron. Pie chart showing the proportion of each type among 99 FN neurons from 3 mice. **g**, **h**, From the FN subpopulation as in (**f**, yellow group), these neuron responses to PC photoactivation by a 10-ms single pulse, 250-ms train, and 500-ms train, showing a time-locked (to the photoactivation offset) post-rebound suppression pattern. **h**, Left: average PSTHs (mean ± s.e.m.) under each condition. Right: comparisons between the timings of rebound-evoked CSs in PCs (see Fig. 4e-h) and the timings of post-rebound suppression in FN neurons. Post-rebound suppression occurred with a fixed time interval after the rebound-evoked CSs (two-sided unpaired *t*-test with Welch’s correction, left to right: *P* = 0.042, 6.40×10^−9^, and 3.11×10^−11^, see numbers of recordings in the panel). Circles and dots represent individual neurons, bars represent mean ± s.e.m. **i**, Illustrative summary depicting that bidirectional modulations of vermal PC SSs induce feedback inhibition in FN neurons via the excitatory FN^E^-IO-PC loop. Left: a simplified excitatory FN^E^-IO-PC loop. Middle: a cascade of neuron responses showing that SS suppression disinhibits FN neurons, thereby driving mcMAO activity and further resulting in CS-induced suppression (red arrowhead) in FN neurons. Right: a cascade of neuron responses showing that SS facilitation initially inhibits FN neurons, resulting in a post-inhibitory rebound facilitation, thereby driving mcMAO activity and a feedback CS-induced post-rebound suppression (red arrowhead) in FN neurons. Source data

**Extended Data Fig. 6:**
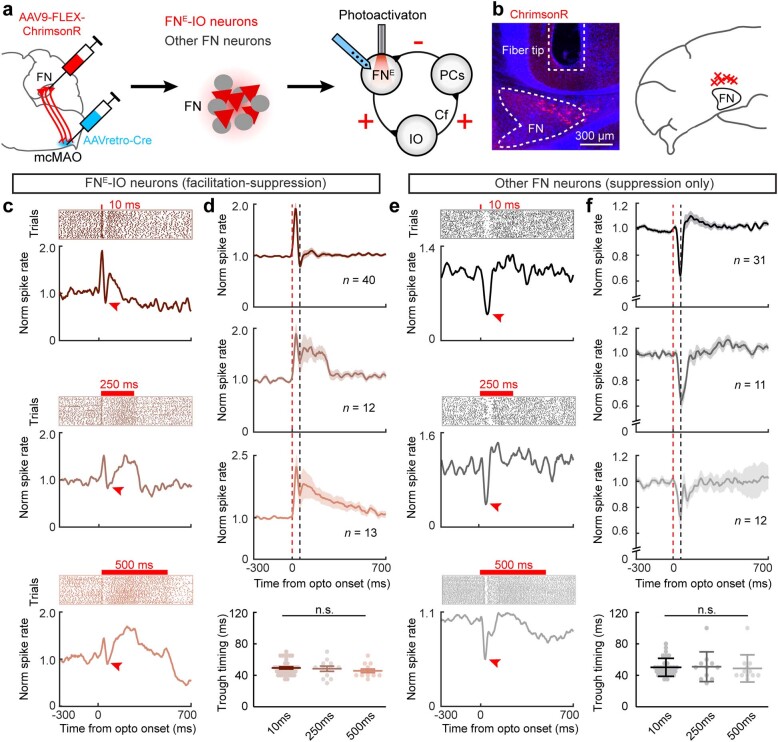
Activating FN^E^-IO neurons induces feedback inhibition on medial cerebellar output. **a**, Experimental schematic of FN recording while photoactivating FN^E^-IO neurons. **b**, Left: example histological image showing ChrimsonR expressing FN^E^-IO neurons and the optic fiber track over the FN. Right: locations of all optic fiber tips from 6 mice (red crosses). **c**, Example raster plots and PSTHs of an opto-tagged FN^E^-IO neuron following stimulation by a 10-ms single pulse, 250-ms train (50 Hz, 50% duty cycle), and 500-ms train (50 Hz, 50% duty cycle), presenting post-faciliatory inhibition (red arrowhead), as an indication of the CS-driven feedback inhibition (see also Extended Data Fig. 5). **d**, Summary of all opto-tagged FN neurons (PSTHs: mean ± s.e.m.) and their post-faciliatory inhibition timing. Red and black dashed lines indicate stimulation onset and suppression trough, respectively. The numbers of cells are shown in the corresponding panels, from 6 mice (dots: individual cells, bars: mean ± s.e.m.). One-way ANOVA with Tukey’s multiple comparisons, *P* = 0.50. **e**, **f**, Same as (**c**, **d**), but for FN neurons only showing putative CS-driven feedback inhibition (red arrowhead), suggesting that activating FN^E^-IO neurons can also drive feedback inhibition on other FN neurons. PSTHs are plotted as mean ± s.e.m. For the comparison of trough timing in (**f**), one-way ANOVA with Tukey’s multiple comparisons, *P* = 0.93; dots: individual cells, bars: mean ± s.e.m. Source data

Synchronized activation of CSs in parasagittal bands of PCs could generate powerful inhibition onto CN neurons^33^. We next investigated the impacts of FN^E^–IO-evoked CS activity on cerebellar outputs. Putative CS-induced inhibition of FN neurons was indeed observed when we bidirectionally manipulated vermal PCs. Photoinhibiting vermal PCs resulted in an initial disinhibition and subsequent postfaciliatory suppression in a group of FN neurons (Extended Data Fig. 5a,b). The FN suppression trough was time-locked to PC photoinhibition onset (Extended Data Fig. 5c,d), consistent with the timing of evoked CSs while photoinhibiting PCs (Fig. 4a–d). Photoactivating vermal PCs induced postrebound suppression in a subpopulation of FN neurons (Extended Data Fig. 5e,f). The FN suppression trough was time-locked to the PC photoactivation offset (Extended Data Fig. 5g,h), in line with the timing of evoked CSs while photoactivating PCs (Fig. 4e–h). Therefore, we speculated that PC modulation activates FN neurons and subsequently drives feedback CSs via the excitatory FN^E^–IO–cortical loop, resulting in a postfaciliatory inhibition pattern in FN neurons (Extended Data Fig. 5i). To test this, we selectively activated FN^E^–IO neurons while recording the surrounding FN neurons (Extended Data Fig. 6a,b). Photo-tagged FN^E^–IO neurons (Methods) indeed presented a postfaciliatory inhibition pattern (Extended Data Fig. 6c,d), which aligned with the timing of evoked CSs (Fig. 4i). Pharmacological inhibition of mcMAO eliminated the postfaciliatory inhibition, resulting in prolonged facilitation in FN neuron activity (Fig. 4j–l). Additionally, a subgroup of FN neurons showed only suppression in response to FN^E^–IO activation (Extended Data Fig. 6e,f), suggesting that feedback CSs, driven by the FN^E^–IO–cortical loop, may inhibit FN neurons across different cerebello-olivary micro modules. Collectively, our results provide systematic evidence that the excitatory FN^E^–IO–cortical loop drives feedback CS activity and shapes medial cerebellar outputs.

### FN^E^–IO neurons project to motor and nonmotor regions

**Fig. 5:**
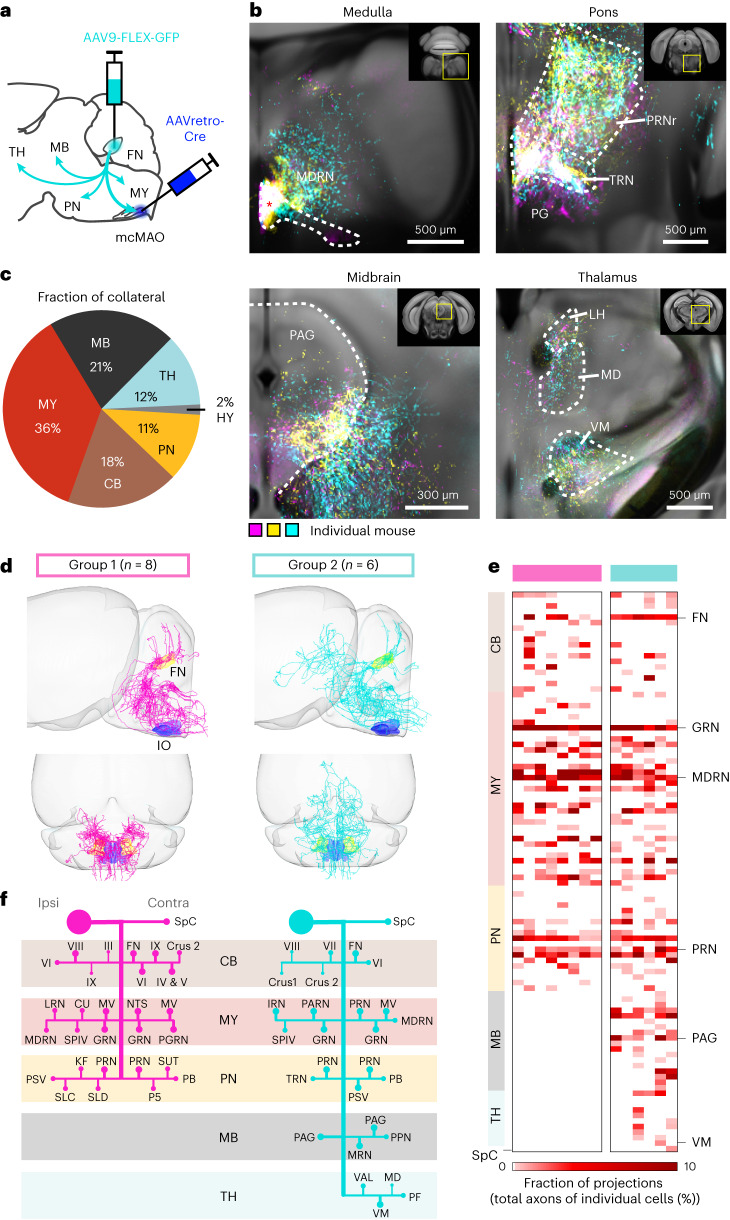
FN^E^–IO neurons send widespread projections to downstream motor and nonmotor regions. **a**, Experimental strategy for tracing FN^E^–IO neuron projections. **b**, Widespread axonal collaterals from FN^E^–IO neurons in the MY, PN, MB and TH. Histological images from three mice (magenta, yellow and cyan indicate individual mice) are aligned to the Allen Mouse Brain CCF. Asterisk in the medulla indicates the mcMAO region, in which AAVretro-Cre was injected. **c**, Brain-wide distribution of the axonal collaterals from FN^E^–IO neurons (*n* = 3 mice). The fraction of collateral is normalized to the total axonal labeling of individual mice. **d**, Overview of FN^E^–IO neuron projections from single-neuron tracing experiments (Methods). Two distinct groups are assigned based on their unique projection patterns—both groups collateralize to the MY, PN and cerebellar cortex, but only neurons in group 2 project to the MB and TH. **e**, Axon density matrix of individual neurons. Each column represents one FN^E^–IO neuron, and each row represents one downstream target. Projection density is normalized to the total number of axons for each cell. Several key regions are highlighted on the right. **f**, Objective visual inspection summarizes the projection motifs for group 1 and group 2 FN^E^–IO neurons. Representative projection targets are denoted by bars and dots with different sizes indicating different densities. CB, cerebellum; MY, medulla; PN, pons; MB, midbrain; TH, thalamus; HY, hypothalamus. Source data

**Extended Data Fig. 7:**
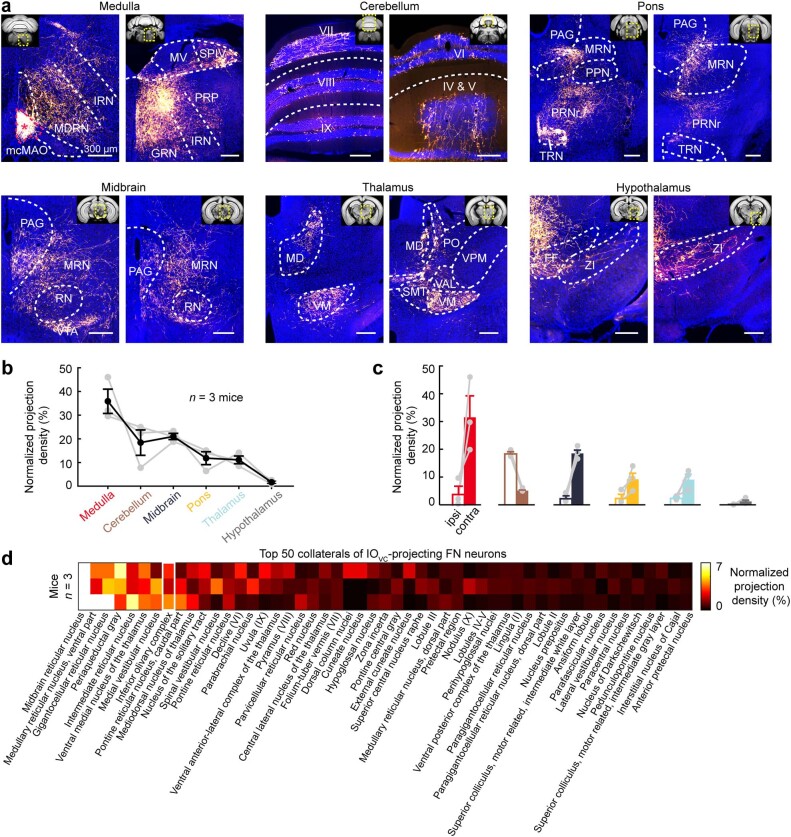
Brain-wide quantification of axonal projection from FN^E^-IO neurons. Related to (Fig. 5a-c). **a**, Example images of different brain regions receiving FN^E^-IO neuron projection. Anterograde AAV9-FLEX-GFP was injected into the left FN and AAVretro-Cre was injected into the contralateral mcMAO to specifically label the FN^E^-IO neurons (red asterisk in the first image). See the tracing schematic in (Fig. 5a). **b**, Fractions of axon collaterals in six major brain regions. Labeling intensity is normalized to the total labeled axons of each mouse. Gray dots: individual mice, black dots and bars: mean ± s.e.m. (*n* = 3 mice). **c**, Comparison of the ipsilateral and contralateral projections in six major brain regions (see color codes in **b**). Gray dots: individual mice, bars: mean + s.e.m. (*n* = 3 mice). **d**, Projection density matrix of the top 50 regions targeted by FN^E^-IO axons. Each row represents one animal. Nuclei are sorted from left to right based on the projecting density. The mcMAO is indicated by a white rectangle. IRN: intermediate reticular nucleus, MDRN: medullary reticular nucleus, MV: medial vestibular nucleus, SPIV: spinal vestibular nucleus, PRP: nucleus prepositus, GRN: gigantocellular reticular nucleus, PAG: periaqueductal gray, MRN: midbrain reticular nucleus, PPN: posterior pretectal nucleus, PRNr: pontine reticular nucleus, TRN: tegmental reticular nucleus, RN: red nucleus, VTA: ventral tegmental area, MD: mediodorsal nucleus of thalamus, VM: ventral medial nucleus of the thalamus, PO: posterior complex of the thalamus, VPM: ventral posteromedial nucleus of the thalamus, VAL: ventral anterior-lateral complex of the thalamus, SMT: submedial nucleus of the thalamus, FF: fields of Forel, ZI: zona incerta. Source data

FN consists of heterogeneous groups of excitatory neurons that project to a large collection of downstream regions and mediate diverse motor and nonmotor functions^20,34^. To illustrate the functional specificity of the FN^E^–IO pathway, we first sought to pinpoint the downstream regions that receive inputs from the FN^E^–IO neurons. We labeled FN^E^–IO neurons by injecting AAV9-FLEX-GFP into the FN and AAVretro-Cre into the contralateral mcMAO region (Fig. 5a). The brain-wide axonal projections were aligned to the Allen Mouse Brain CCF and quantified using a customized analysis pipeline^35^ (Methods). Apart from the dense labeling in the mcMAO, extensive axonal collaterals were found in over 200 regions throughout the thalamus, hypothalamus, midbrain, pons, cerebellum and medulla (Fig. 5b,c and Extended Data Fig. 7). The majority of axonal labeling was distributed contralateral to the FN, except for the cerebellar nucleo-cortical projection^36^, which spanned both sides (Extended Data Fig. 7c). In line with the general FN projection pattern^37^, over 60% of the total collaterals were distributed in the brainstem. Within the thalamus, the ventral medial (VM) and medial dorsal (MD) nuclei were labeled, suggesting potential roles of the FN^E^–IO neurons in motor planning and other cognitive functions^20,38^. We confirmed the diverse collateralization of the FN^E^–IO neurons by selectively labeling the FN subpopulations that project to the following different downstream regions: the medullary reticular nucleus, VM, pontine reticular nucleus (PRNr) and periaqueductal gray (PAG), showing prominent axonal labeling in the mcMAO regions (Supplementary Fig. 6). Moreover, FN^E^–IO collaterals were also observed in the contralateral spinal cord regions C1 to T1 (Supplementary Fig. 7). Therefore, our results reveal extensive projection patterns of the FN^E^–IO neurons, suggesting their involvement in diverse motor and nonmotor functions.

**Extended Data Fig. 8:**
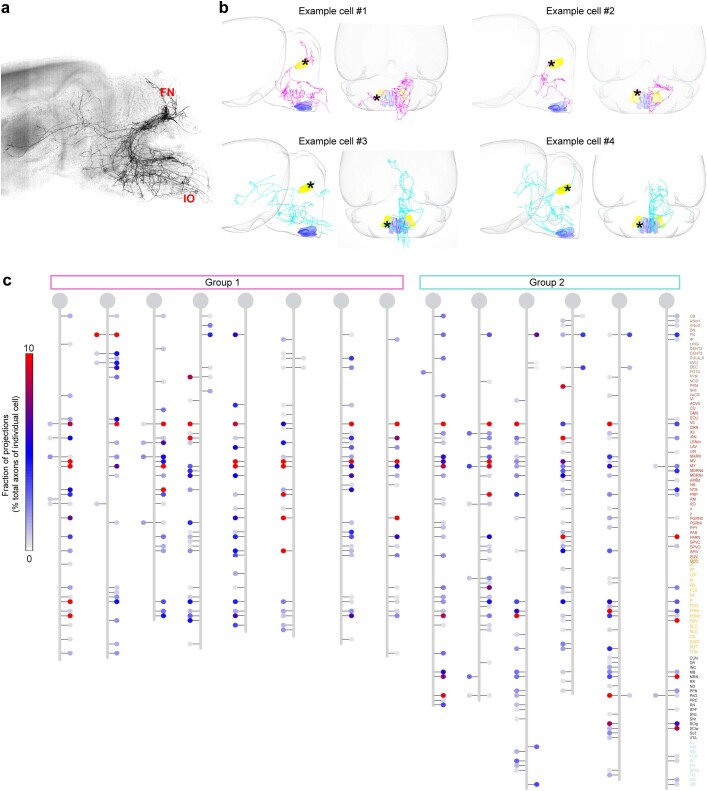
Single-cell projection patterns of FN^E^-IO neurons. Related to (Fig. 5d,e). **a**, Sagittal image of the maximum density projection showing FN^E^-IO projection patterns using the fMOST approach (see Methods). A widespread distribution of FN axons is observed across different brain regions. See the tracing strategy in (Fig. 5a). **b**, Projection maps of four FN^E^-IO single neurons. Axon projection was registered to and rendered in the Allen Mouse Brain CCF. Asterisks indicate the somata of FN^E^-IO neurons (yellow structure), and the IO is marked in blue. Magenta neurons mainly project to the medulla, cerebellar cortex, and pons. Cyan neurons project to the medulla, cerebellar cortex, pons, midbrain, and thalamus. **c**, Comparative projection map of all FN^E^-IO single neurons. The connectivity with downstream targets is represented by bars and dots on the left (ipsilateral projection) and right (contralateral projection). Colors in the dots indicate projection density, normalized to the total terminations of each cell. Detailed downstream targets are listed on the right.

To further dissect the detailed FN^E^–IO neuron projectome, we sparsely labeled these neurons and used the fluorescence micro-optical sectioning tomography (fMOST) technique to map their whole-brain projection at single-axon resolution^39^ (Methods). In total, 14 FN neurons were identified as FN^E^–IO neurons by the presence of axonal terminals in the mcMAO regions (Extended Data Fig. 8a). Closer inspection revealed two groups of FN^E^–IO single neurons with specific projection patterns (Fig. 5d and Extended Data Fig. 8b). Neurons in both groups terminate their axons in the cerebellar cortex, pons, medulla and spinal cord. In addition, neurons in group 2 extended their axons into the midbrain and thalamic regions (Fig. 5e and Extended Data Fig. 8c). Overall, the single-neuron tracing results confirm the widespread projections of the FN^E^–IO neurons and further uncover two stereotypical projection motifs (Fig. 5f).

### Activation of FN^E^–IO neurons elicits directional movements

**Fig. 6:**
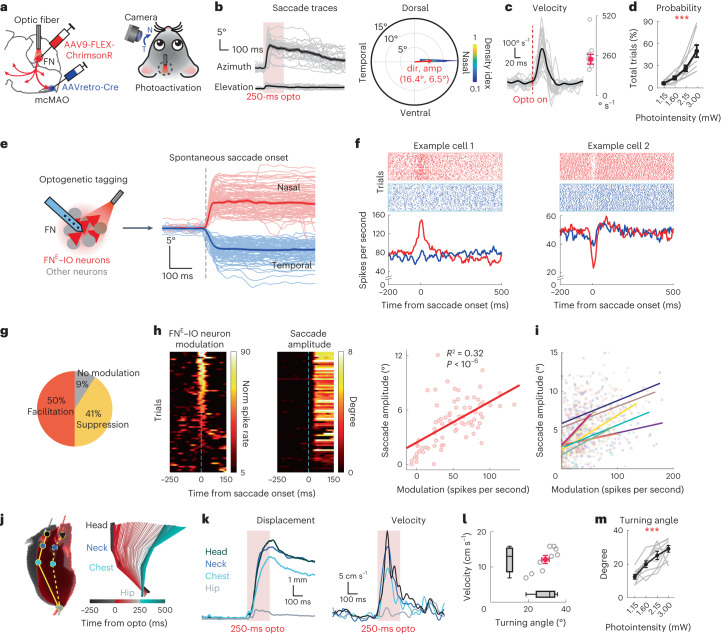
Activation of FN^E^–IO neurons elicits directional saccadic and upper body movements. **a**, Photoactivation of FN^E^–IO neurons while monitoring ipsilateral eye movements (N for nasal and T for temporal). **b**, Left: representative evoked saccades (gray, individual trials; black, mean ± s.e.m.). Right: polar plot indicating the directions and amplitudes of evoked saccades (*n* = 207 trials from nine mice). The distribution of all saccades is presented as the density colormap. Red arrow indicates the average saccade direction and amplitude. **c**, Evoked saccade velocity from the same mouse in **b** and summary of peak velocity from nine mice (right-hand panel, red dot and bars represent mean ± s.e.m.). **d**, Evoked saccade probabilities following increasing photoactivation intensities (*n* = 9, one-way ANOVA with repeated measures, *P* = 0.0005). Dots and bars represent mean ± s.e.m. **e**–**i**, FN^E^–IO neurons modulate spontaneous saccade. **e**, Optogenetic identification and recording of FN^E^–IO neurons during spontaneous saccade (Methods). Lighter traces, individual saccades; darker traces, mean ± s.e.m. **f**, Example neurons recorded during spontaneous saccade (see saccade traces in **e**), showing facilitation and suppression in response to nasal saccade. **g**, Fraction of neuron modulation in response to nasal saccade (*n* = 22 neurons from four mice). **h**, Trial-by-trial correlation between neuron activity and saccade amplitude from an example recording. Trials in the heatmaps are sorted based on the saccade amplitudes. Right: scatter plot of all trials and curve fitting (linear regression model, *P* = 1.1 × 10^−7^) from the same recording. **i**, Summary for all FN^E^–IO neurons that show significant activity-saccade amplitude correlation (*n* = 10). **j**, Left: overlay of two snapshots of a freely moving mouse, illustrating the body postures before (black) and after (red) 250-ms photoactivation. Right: serial plots of hip-chest-neck-head positions over time, illustrating movement trajectory before (black), during (red) and after (turquoise) photoactivation. **k**, The corresponding displacement and velocity for **j**. **l**, Summary of peak velocities and turning angles of photoactivated movements from nine mice. Red dot and bars represent mean ± s.e.m.; box-whisker plots indicate the median, interpreted quartiles and range. **m**, Turning angles positively correlate with the photoactivation intensities (*n* = 9 mice, one-way ANOVA with repeated measures, *P* = 0.0001). Dots and bars represent mean ± s.e.m. Source data

**Extended Data Fig. 9:**
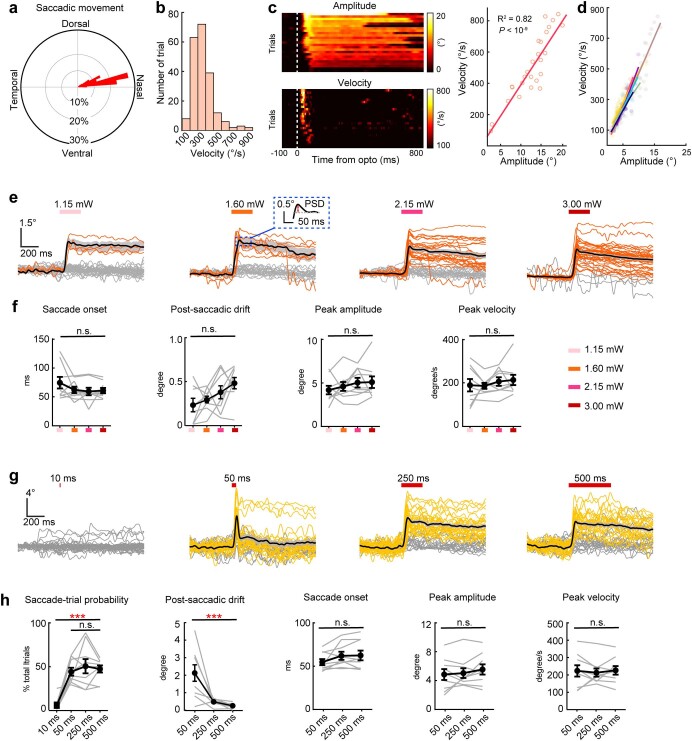
Optogenetic activation of the FN^E^-IO neurons drives saccade. Related to (Fig. 6a-d). **a**, Polarhistogram of the saccade movement directions following 250-ms photoactivation (50 Hz, 50% duty cycle, 3.00 mW). Unilateral activation of FN^E^-IO neurons drives nasal saccade of ipsilateral eye. Number of trials in each bin is normalized to the total saccade trials (*n* = 207 from 9 mice), bin size = 6.4°. **b**, Histogram of the peak velocities of all evoked saccades. **c**, Trial-by-trial correlation between the saccade amplitude and velocity. Left: heatmaps showing saccade amplitudes (upper) and velocities (lower) of a representative mouse. Each row represents one trial, and all trials are sorted based on the peak velocities. The dashed line indicates photoactivation onset. Right: scatter plot of all saccade trials (circles) and the curve fitting (linear regression model, *P* = 3.5×10^−10^) from the same mouse (see R^2^ value in the panel). **d**, Group summary from 9 mice indicating consistent trial-by-trial correlations between evoked saccade velocity and amplitude. **e**, Representative eye movement traces following 250-ms photoactivation with graded intensities in an example mouse. Orange traces are identified as photo-evoked saccades (see Methods); gray traces are unresponsive trials; black traces indicate the average saccadic movement of each condition (mean ± s.e.m.). Post-saccadic drift (PSD in the blue shaded-line rectangle) is defined as the movement deviation from the saccade peak to the position at 50 ms after the peak. The probabilities of evoked saccades increase as a function of photoactivation intensities (see summary for all mice in Fig. 6d). **f**, Summary of the saccade onset, PSD, amplitude, and velocity for different photoactivation intensities. One-way ANOVA with Tukey’s multiple comparisons, *n* = 9 mice, *P* = 0.18, 0.06, 0.22, and 0.37 for each comparison. Dots and bars represent mean ± s.e.m. **g**, **h**, Same as (**e**, **f**), but for eye movements following different photoactivation durations. Yellow traces are identified as photo-evoked saccades; gray traces are unresponsive trials; black traces are the average saccade movements (mean ± s.e.m.). **h**, The probability of evoked saccade following 10-ms photoactivation was minimal compared to the other conditions (one-way ANOVA with Tukey’s multiple comparisons, *n* = 9 mice, *P* = 1.72×10^−6^). There was no significant difference in saccade probabilities among the 50-ms, 250-ms, and 500-ms photoactivations (*P* = 0.51). The kinematics of evoked saccades are dependent on the photoactivation duration. Photoactivation of 50 ms evokes distinct saccadic movements which rapidly return to the initial eye position, resulting in larger PSDs but comparable onsets, amplitudes, and velocities to those driven by 250-ms and 500-ms photoactivations (*P* = 0.0041, 0.078, 0.32, and 0.26 for each comparison). Dots and bars represent mean ± s.e.m. Source data

The medial cerebellum is involved in oculomotor, axial and proximal motor control^20,40^. FN^E^–IO neurons project to the PRNr, gigantocellular reticular nucleus (GRN) and spinal cord (Fig. 5), implying their involvement in saccadic eye movements^41^ and head/neck movements^42^. To test this, we first activated the FN^E^–IO neurons and monitored eye movements in head-restrained animals (Fig. 6a). Transient stimulation consistently elicited saccadic eye movements along the horizontal axis (azimuth) toward the nasal direction, with minimal vertical elevation (Fig. 6b, Extended Data Fig. 9a and Supplementary Video 1). The photo-evoked saccades showed an average velocity of 213.2 ± 23.7° s^−1^ and amplitude of 6.5 ± 0.7° (Fig. 6c and Extended Data Fig. 9b). The peak velocity and amplitude were linearly correlated on a trial-by-trial basis (Extended Data Fig. 9c,d), consistent with the kinematics of triggered and self-generated saccades in rodents^43,44^. We graded the optogenetic stimulation intensities and durations to further clarify the causal relationship between FN^E^–IO neuron activity and saccadic eye movements. Increasing photoactivation intensities substantially enhanced the probabilities of the evoked saccades (Fig. 6d and Extended Data Fig. 9e,f). Different durations of photoactivation elicited saccadic movements with comparable onsets, amplitudes and peak velocities, except for the 10-ms condition (Extended Data Fig. 9g,h). Notably, the short activation (50 ms) drove the initial saccade but did not maintain the stable gaze shift, resulting in a larger postsaccadic drift of approximately 2° (Extended Data Fig. 9g,h). To further establish causality, we investigated whether FN^E^–IO neurons are active during spontaneous saccades. We identified the photo-tagged FN^E^–IO neurons and subsequently recorded their activities during spontaneous saccadic movements of the ipsilateral eye (Fig. 6e). Most (20/22) of the photo-tagged FN^E^–IO neurons had significant modulations in response to the horizontal saccades. The modulation is stronger toward the nasal direction (Fig. 6f,g). Moreover, a subpopulation of these neurons (9/22) showed a significant trial-by-trial correlation between their modulations and saccade amplitudes (Fig. 6h,i). These data collectively provide evidence for the involvement of FN^E^–IO neurons in modulating saccadic eye movements.

**Extended Data Fig. 10:**
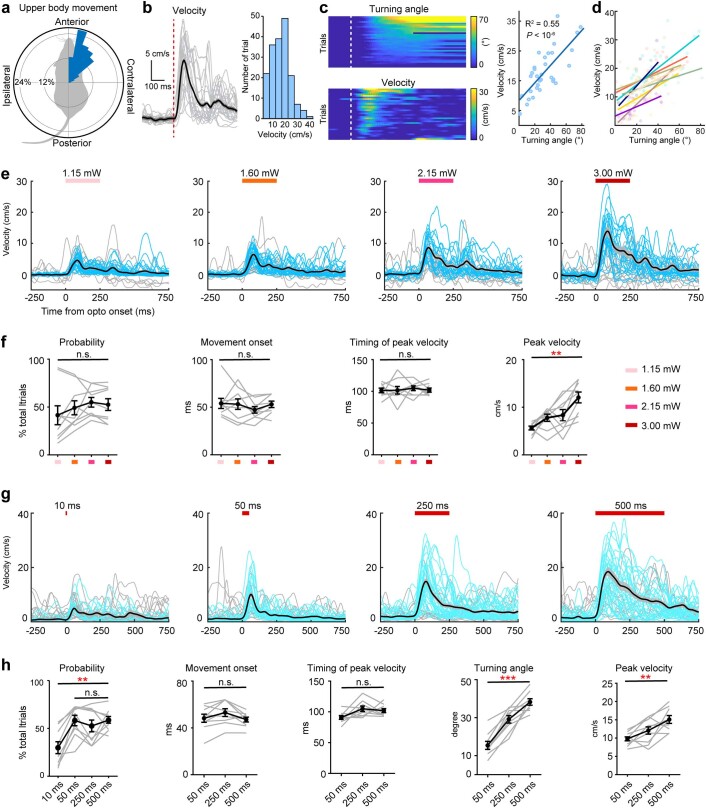
Optogenetic activation of FN^E^-IO neurons drives upper body movements. Related to (Fig. 6e-h). **a**, Polarhistogram of the upper body turning direction following 250-ms photoactivation (50 Hz, 50% duty cycle, 3.00 mW). Unilateral activation of the FN^E^-IO neurons drives upper body turning toward the contralateral direction. The number of trials in each bin was normalized to the total trials in the polarhistogram (*n* = 216 from 9 mice), bin size = 9.2°. **b**, Left: velocities of upper body movements driven by FN^E^-IO activation from an example mouse. Red dashed line indicates the photoactivation onset. Right: distribution of the peak velocities from all mice (*n* = 216 trials). **c**, Trial-by-trial correlation between the upper body turning angle and the movement velocity. Left: heatmaps of the turning angle (upper) and the movement velocity (lower) from a representative mouse. Each row represents one trial, and all trials are sorted based on the turning angle. Dashed line indicates photoactivation onset. Right: scatter plot of all evoked upper body movements and the curve fitting (linear regression model, *P* = 6.8×10^−7^) from the same mouse. **d**, Summary of the turning angle-velocity correlations of all 9 mice. **e**, Movement velocities in response to 250-ms photoactivation with graded intensities. Blue traces indicate trials with detectable movements (see Methods); gray traces indicate nonresponsive trials; black traces indicate the average turning velocities. **f**, Summary of the evoked probabilities, movement onset, timing of peak velocity, and peak velocity in response to graded photoactivation intensities. Friedman test with paired Dunn’s multiple comparisons, *n* = 9 mice, *P* = 0.51, 0.11, 0.90, and 0.0012 for each comparison. Dots and bars represent mean ± s.e.m. **g**, **h**, Same as (**e**, **f**), but for the upper body movement following photoactivations with graded durations (3.0 mW, durations indicated in each panel). Cyan traces indicate trials with detectable photo-evoked movements. **h**, The evoked probability of movement in response to 10-ms photoactivation was minimal compared to other conditions (Friedman test with paired Dunn’s multiple comparisons, *n* = 9 mice, *P* = 0.0022). There was no significant difference in the evoked probabilities of movements among the 50-ms, 250-ms, and 500-ms photoactivations (*P* = 0.57). There was no difference in the movement onset or the timing of peak velocity (*P* = 0.057 and 0.28, respectively), but the maximum turning angle (*P* = 1.35×10^−4^) and velocity (*P* = 0.0029) positively correlated with the photoactivation durations. Dots and bars represent mean ± s.e.m. Source data

Next, we examined the impacts of activating FN^E^–IO neurons on the body movements of freely moving mice. Selective optogenetic activation of these neurons evoked rapid turning of the upper body toward the contralateral side (Fig. 6j, Extended Data Fig. 10a and Supplementary Video 2). Attempted movements were also observed in head-fixed animals (Supplementary Fig. 8), suggesting that the head turning driven by FN^E^–IO neurons is not strictly context-dependent but an innate behavior. The evoked movements showed an average head-neck turning angle of 29.1 ± 2.0° and a peak velocity of 12.1 ± 0.1 cm s^−1^ (Fig. 6k,l and Extended Data Fig. 10b). The peak velocity and turning angle were linearly correlated on a trial-by-trial basis (Extended Data Fig. 10c,d), similar to the photo-evoked saccade. Moreover, both the peak velocity and turning angle increased as functions of the stimulation intensity and duration (Fig. 6m and Extended Data Fig. 10e–h). Photoactivation of another FN population, the GABAergic neurons, induced neither eye movement nor upper body movement (Supplementary Fig. 9). These results collectively demonstrated that synchronized activation of FN^E^–IO neurons is necessary and sufficient to drive rapid movements.

### FN^E^–IO–cortical modules contribute to precise motor control

**Fig. 7:**
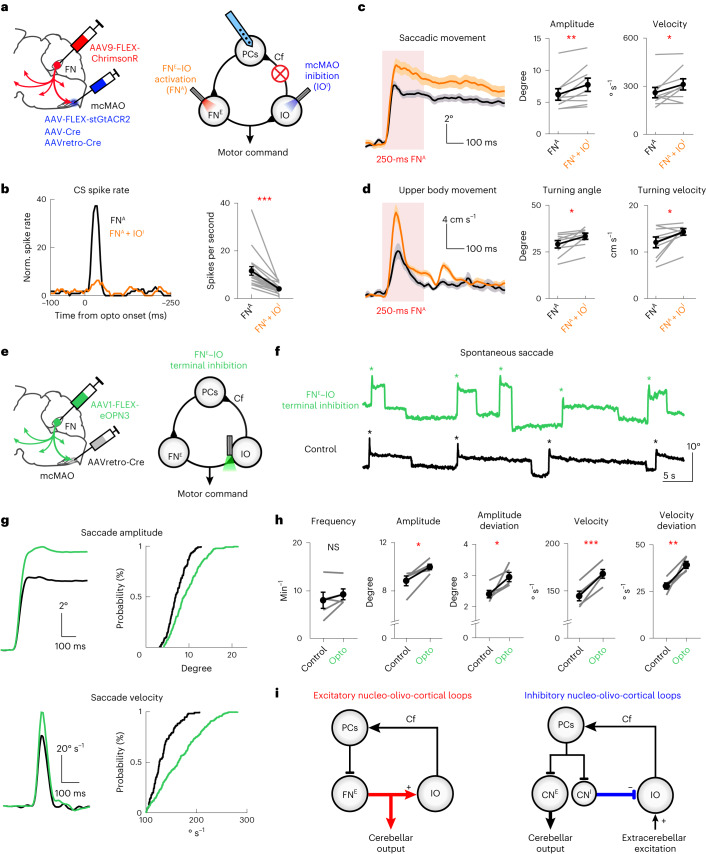
Feedback CS activity driven by FN^E^–IO–cortical loop is essential for precise motor control. **a**, Schematics showing simultaneous photoactivation of ChrimsonR-expressing FN^E^–IO neurons (FN^A^, 50 Hz, 50% duty cycle and 3.0 mW) and photoinhibition of stGtACR2-expressing mcMAO neurons (IO^I^, 50 Hz, 50% duty cycle and 2.4 mW). See detailed optogenetic protocols in ‘Methods’. **b**–**d**, Comparison of FN^A^-evoked CS probability, saccadic movement and upper body movement, with and without mcMAO inhibition (two-sided Wilcoxon matched-pairs signed rank test). **b**, Vermal PC recordings showing decreased FN^A^-evoked CS activity by mcMAO inhibition. Left: evoked CS activity of an example PC. Right: maximum spike rates of FN^A^-evoked CS for all PCs (*n* = 18 cells from three mice, *P* = 7.6 × 10^−6^). Dots and bars represent mean ± s.e.m. **c**, Left: example FN^A^-driven saccade before and after mcMAO inhibition. Traces are plotted as mean ± s.e.m. Right: group summary showing increased saccadic amplitude and velocity after mcMAO inhibition (*n* = 9 mice, *P* = 0.0078 and 0.027). Dots and bars represent mean ± s.e.m. **d**, Same as **c**, but for the FN^A^-evoked upper body movements before and after mcMAO inhibition (*n* = 9 mice, *P* = 0.027 for both comparisons). **e**, Schematics of long-term inhibition of FN^E^ terminals on mcMAO during spontaneous saccades (lasting inhibition, 50 Hz, 50% duty cycle and 2.5 mW). **f**, Raw traces of spontaneous saccadic movements from an example mouse following FN^E^–IO terminal inhibition (green) and control (black) conditions. An asterisk indicates nasal saccades. **g**, Average trace and accumulative distribution of saccade amplitude and velocity from the same mouse in **f**. **h**, Effects of FN^E^–IO terminal inhibition on spontaneous saccade frequency, amplitude, amplitude deviation, velocity and velocity deviation (*n* = 5, two-sided paired *t*-test, *P* = 0.209, 0.025, 0.041, 0.0010 and 0.010). Gray dots represent individual mice, and black dots and bars represent mean ± s.e.m. **i**, Summary of the excitatory and inhibitory cerebellar nucleo-olivo-cortical motifs for motor control. Excitatory projections are indicated by arrows and inhibitory projections are indicated by flat-end lines. Source data

What is the contribution of the feedback CSs to the FN^E^–IO-driven movements? To address this question, we simultaneously activated ChrimsonR-expressing FN^E^–IO neurons and inhibited GtACR2-expressing mcMAO neurons (Fig. 7a). This configuration reliably blocked the FN^E^–IO-driven feedback CSs in vermal PCs (Fig. 7b). As shown in our FN recordings (Fig. 4j–l), pharmaceutical inhibition of mcMAO eliminated CS-induced inhibition in FN neurons, resulting in prolonged activation of FN outputs. Therefore, we speculate that eliminating feedback CSs (mcMAO photoinhibition) would enlarge FN excitation (FN^E^–IO photoactivation), resulting in excessive saccadic and upper body movements. Indeed, 250-ms photoactivation of FN^E^–IO neurons, coinciding with the photoinhibition of the feedback CSs, induced substantially larger velocities and amplitudes for both saccade and upper body movements, without influencing the movement timing (Fig. 7c,d and Supplementary Fig. 10a). We next shortened the FN^E^–IO activation to mimic the FN modulation pattern during natural movement (Supplementary Fig. 10b,c). A 50-ms photoactivation of the FN^E^–IO neurons, while inhibiting the feedback CSs, consistently resulted in movements with larger velocities and amplitudes (Supplementary Fig. 10b–d).

Because FN^E^–IO neurons modulate their activities during spontaneous horizontal saccades (Fig. 6e–i), we next examined the functional role of the FN^E^–IO pathway in modulating volitional saccadic movements. Under control condition (Methods), mice had a nasal saccade frequency of 8.1 ± 1.7 events per minute, mean amplitude of 8.8 ± 0.4° and mean velocity of 144.7 ± 5.1° s^−1^. Tonic optogenetic inhibition of the FN^E^ terminals in IO using a potent inhibitory opsin eOPN3^45^ (Fig. 7e) substantially increased the spontaneous saccade amplitude and velocity of the same animals, whereas the saccade frequency was unaltered (Fig. 7f–h). FN^E^–IO terminal inhibition also resulted in larger variabilities of the saccadic amplitude and velocity, revealing the involvement of the FN^E^–IO pathway in modulating the vigor and precision of volitional saccadic movements (Fig. 7g,h). Taken together, our data illustrate the crucial roles of the excitatory FN^E^–IO–cortical loop in motor control.

## Discussion

In contrast to the canonical inhibitory nucleo-olivary wiring diagram, we demonstrate that the excitatory FN–IO pathway drives CS firing in vermal PCs and shapes the cerebellar motor command that controls rapid movements. Based on these findings, we propose two distinct nucleo-olivo-cortical motifs in the cerebellum (Fig. 7i), which could collectively, yet distinctly, finetune the motor commands to achieve high spatiotemporal precision in movements.

The fundamental differences in the circuitry design possibly lead to the functional specialization of different cerebellar modules^5^. The medial cerebellum is critically involved in controlling axial and proximal movements, such as saccadic, orofacial and upper body movements, that require high velocity and accuracy^10,32,40^. A commonly accepted hypothesis suggests that the accuracy of saccade endpoint is incorporated by an internal feedback signal processed through the cerebellum, which corrects it online^46^. Consistent with this internal model theory, an excitatory CN–IO connection is computationally optimal for providing a short-latency feedback signal of the motor outputs and calibrating ongoing movements^3,47^. In line with this notion, we report that the FN^E^–IO neurons are equipped to activate widespread downstream premotor regions while simultaneously providing an internal feedback CS signal (Fig. 7i, left motif). This cerebellum-driven CS signal could encode a copy of the FN motor command (an efference copy from the cerebellar perspective) and directly finetune movements^1^. In contrast, the excitatory output of the intermediate/lateral cerebellar modules diverges from the inhibitory nucleo-olivary pathway (Fig. 7i, right motif) and suppresses CS activity once activated. Therefore, the intermediate/lateral cerebellar modules may implement distinct computational mechanisms and operate in different spatiotemporal domains. Indeed, the IO regions that target the intermediate/lateral cerebellar cortices receive prominent excitatory inputs from the mesodiencephalic junction, an extracerebellar hub region that integrates diverse cerebral information^48^. We postulate that climbing fiber inputs to the intermediate/lateral cerebellar modules convey diverse cerebral information and are likely subject to motor control mediated by the inhibitory nucleo-olivary pathway^49^.

Within the forward model of motor control framework, the cerebellum is considered to generate predictions for future movements^1,50,51^. Such computation thus requires IO neurons to compare the extracerebellar motor command and cerebellar prediction and to generate CSs when these two mismatches. The error/instructive signals conveyed by climbing fiber inputs are thought to mediate cerebellar plasticity using supervised learning^52–54^. The inhibitory CN–IO projection is particularly useful for preventing saturation or causing extinction^27,55^. Our study suggests new computational principles for cerebellar learning. The FN^E^–IO pathway is designed to drive precisely timed feedback CSs, crucial for instructing long-term plasticity in the cerebellar cortical neurons and cerebellar learning^55^. Given that the FN neurons have relatively high intrinsic firing rates, neurons in the mcMAO are likely to function as high-pass filters that translate specific FN activity patterns into well-timed CSs during learning. Under this scenario, cerebellum-driven internal feedback could be directly integrated into the same cerebellar module, or even into the same PC, and induce plasticity within the loop. Such circuit design might be most efficient for providing feedback information regarding the consequence of its computation and thereby optimizing learning via backpropagation^47^. This form of feedback information may be similar to a prediction error signal during reinforcement learning^56,57^. Therefore, we hypothesize that the FN^E^–IO modules are in principle well-equipped to generate internal models, by performing not only supervised learning but also reinforcement learning^58–60^. Below we speculate two plausible scenarios that the FN^E^–IO modules could contribute to cerebellar learning. First, the FN^E^–IO modules could contribute to enhancing the medial cerebellar outputs during learning. When the FN^E^–IO neurons are activated, the increased climbing fiber input to the cerebellar cortex may be considered a form of positive prediction error. Synaptic plasticity in the cerebellar cortex, governed by this positive prediction error, may loop back to the same FN neurons and further strengthen the desired cerebellar outputs during subsequent learning (Supplementary Fig. 11a). Such positive feedback loop is consistent with the gradual emergence of CSs during saccadic adaptation^17^. Another possibility is that the feedback CSs might carry sensory prediction errors when the expectation computed by the cerebellum does not match with the actual sensory feedback^61^. Assuming FN functions as a comparator between the cerebellar output and the sensory feedback, it is likely that the mismatch activates FN neurons and subsequently generates feedback CSs via the FN^E^–IO–PC pathway. Using a rich repertoire of synaptic plasticity rules^55^, instructed by this CS signal, the cerebellum could finetune the internal model for motor adaptation. Indeed this conjecture is consistent with the finding in monkeys that FN neurons display strong activation after the introduction of perturbation and gradually decline during the adaptive head turning^61^ (Supplementary Fig. 11b). Interestingly, the excitatory and inhibitory FN–IO modules segregate the cerebellar cortical regions (Fig. 2). Having parallel access to both supervised learning and reinforcement learning may vastly enhance the capacity of cerebellum learning.

The current theory also highlights the impacts of cerebellar outputs on olivary neuron synchrony. IO neurons are electrically coupled via gap junctions, which control the subthreshold voltage oscillation and synchronize CS discharges in a collection of parasagittal PCs^62,63^. While learning a directional forelimb movement task, PCs in lobule V–VI showed highly synchronized CS activity across cerebellar cortical regions^49^. These regions largely overlap with the vermal regions that project to the FN^E^–IO neurons and receive mcMAO climbing fiber inputs (Fig. 2), suggesting a potential role of the FN^E^–IO–cortical loop in regulating CS synchrony during skilled movements. The excitatory FN neurons have intrinsic spike rates at an average of 60 Hz^64^ and can bidirectionally modulate their activity in a broad range of frequencies during motor learning. A direction for future study would be to determine how neural dynamics of the FN^E^–IO pathway regulates CS synchronicity and supervise cerebellar motor learning.

It has been recently suggested that the stereotyped CN neurons are duplicated during phylogeny to form new subnuclei, and their genetic profiles and projection patterns are modified accordingly to achieve particular functions^37^. The repetitive inhibitory CN–IO pathway is consistent with this ‘duplication-and-divergence’ framework^37^. However, the excitatory CN–IO pathway is confined to FN, the phylogenetically oldest CN^37,65^, presenting a curious case for evolutionary neurobiology. Indeed, although all three nuclei innervate a large collection of brain regions, the IN and DN projection patterns are more identical than the FN projection patterns^34,37^. Therefore, the medial cerebello-olivery module possesses a unique circuitry that distinguishes itself from the rest of the cerebellum.

Apart from its role in motor control, the functional significance of the FN^E^–IO pathway in nonmotor and cognitive behaviors remains to be determined. The contribution of the cerebellar vermis and FN to nonmotor and cognitive functions has been increasingly understood in recent years^20,38,40,49^. FN is reciprocally connected to the frontal cortex and the cortico-cerebellar loop is critically involved in constructing preparatory activity before planned movements^38^. In a learned task that requires high spatiotemporal curacy of neural computation in the vermal cerebellum, the FN^E^–IO pathway is likely engaged to generate timed feedback CSs during motor planning. Here we provide evidence that the FN^E^–IO modules target multiple downstream regions that are involved in nonmotor and cognitive functions (Fig. 5). Considering the vast convergence and divergence of the cerebellar circuits^34,37^, it is likely that multiple nucleo-olivo-cortical modules (Fig. 7i) operate in synergy to mediate higher cognitive functions.

## Methods

### Mice

All animal experiments were approved by the institutional animal welfare committee of Erasmus MC in accordance with the central authority for scientific procedures on animal guidelines. Wild-type C57BL/6J (000664), transgenic *VGluT2*-ires-Cre (016963), *Gad2*-ires-Cre (010802), *L7*-Cre (004146), Ai27D (012567) and R26-LNL-GtACR1-Fred-Kv2.1 (033089) mice were obtained from Jackson Laboratory, and *GlyT2*-ires-Cre mice were originally obtained from S. Dieudonné (Institut de Biologie de l’ENS). We used both male and female mice in this study. All mice in this study were 8–16 weeks old and housed in a 12-h light/12-h dark cycle with food and water ad libitum. All experiments were performed during the light cycle. The ambient housing temperature was maintained at approximately 25.5 °C with 40–60% humidity. We crossed the *L7*-Cre mice with the Ai27D mice to express the excitatory opsin ChR2 in PCs (*L7*Cre-Ai27) and crossed the *L7*-Cre mice with the R26-LNL-GtACR1-Fred-Kv2.1 mice to express the inhibitory opsin GtACR1 in PCs (*L7*Cre-GtACR1).

### Viral vectors

For FN bulk tracing, we used adeno-associated virus AAV9-hSyn-RFP (UNC Vector Core). For cell-type and pathway-specific tracing, we used AAV9-CAG-FLEX-tdTomato (UNC Vector Core), AAV9-CAG-FLEX-GFP (UNC Vector Core) and AAV9-hSyn-FLEX-mGFP-2A-Synaptophysin-mRuby (Charité Viral Core Facility). For transneuronal retrograde tracing, we used AAVretro-CMV-FLEX-TVAmCherry-2A-oG (Charité Viral Core Facility) and rabies virus RV-CMV-EnvA-ΔG-eGFP (Charité Viral Core Facility). For optogenetic manipulation, we used AAV9-Syn-FLEX-ChrimsonR-tdTomato (UNC Vector Core), AAV1-hSyn1-SIO-stGtACR2-FusionRed (Addgene) and AAV1-hSyn-SIO-eOPN3-mScarlet (Addgene). For expressing Cre recombinase in different experiments, we used retrograde AAVretro-CAG-Cre (UNC Vector Core), transneuronal anterograde AAV1-CMV-Cre-GFP (Addgene) and AAV5-hSyn-Cre-GFP (UNC Vector Core). All viral vectors were aliquoted and stored at −80 °C until used.

### Surgical procedures

Mice were anesthetized with 5% isoflurane for induction and 2.5% for maintenance. Mice were fixed on a mouse stereotaxic surgical plate (David Kopf Instruments) with eyes covered by DuraTears (Alcon Laboratories), and body temperature was maintained at 37 ± 0.5 °C during the operation. Bupivacaine (4 mg kg^−1^) was administrated intraperitoneally after surgery. For intracranial viral injection, the skull was exposed and the head position was adjusted to level the bregma and lambda. We then opened a small cranial window (*Φ* = 300 μm) according to the stereotaxic coordinates for different brain regions (Supplementary Table 1). A glass capillary (tip opening *Φ* = 5–8 μm) was lowered in the targeted region, and viral vector (20–50 nl) was slowly injected. The glass capillary was left in the injection site for approximately 5 min before slowly retracting from the brain. The incubation time for viral transfection in different experiments is as follows: 4 weeks for cell-type/pathway-specific tracings; 6 weeks for transneuronal tracing of climbing fibers; 4 weeks for rabies helper virus and 7 d for rabies incubation; 4–5 weeks for optogenetic experiments.

For optogenetic manipulation of the FN neurons, a 2-mm long optical fiber (*Φ* = 200 μm; 0.22 NA, Thorlabs) was inserted through a small cranial window (*Φ* = 300 μm) over the FN and chronically fixed to the skull with dental cement (Charisma, Heraeus Kulzer). For optogenetic manipulation of the mcMAO neurons or FN axons in the mcMAO, the same operation was performed except that a 5-mm long optic fiber (*Φ* = 200 μm; 0.22 NA, Thorlabs) was implanted over the mcMAO. For simultaneous manipulation of FN and mcMAO, a 2.5-mm long optical fiber (*Φ* = 200 μm; 0.22 NA, Thorlabs) was inserted with an angle of 40°, relative to the anteroposterior axis, over the FN to ensure enough space for the fiber implantation over the mcMAO. For the delivery of muscimol in mcMAO, a 5-mm long guide cannula (*Φ* = 410 μm, RWD Life Science) was chronically implanted over the mcMAO region. A dummy cannula (*Φ* = 210 μm, RWD Life Science) was used for daily protection, and an internal cannula was used for muscimol administration (*Φ* = 200 μm, RWD Life Science). For head fixation, a 5.5 × 4.0 mm custom-made brass pedestal was attached to the skull with dental cement. For in vivo electrophysiology, a cranial window (*Φ* = 2.0 mm) was made on the skull over the recording sites. We built a chamber around the craniotomy with dental cement and sealed it with silicone glue (Picodent Twinsil) for protection.

### Immunofluorescence histology

Mice were deeply anesthetized with an intraperitoneal injection of pentobarbital sodium solution (50 mg kg^−1^) and perfused transcardially with saline, followed by 4% paraformaldehyde (PFA) in 0.1 M phosphate buffer (PB; pH 7.4). Brains were removed immediately and postfixed overnight in 4% PFA and 0.05 M PB at 4 °C. Fixed brains were placed in 10% sucrose overnight at 4 °C and embedded in 12% gelatin and 10% sucrose. After fixation in 10% formalin and 30% sucrose overnight at 4 °C, serial coronal sections were cut using a microtone (Leica Biosystems, SM2000R) at 50 µm and collected in 0.05 M PB. For immunofluorescence, sections were incubated subsequently with primary and secondary antibodies (see titrations below). All antibodies were titrated for working solution with 2% normal horse serum, 0.5% triton and 0.1 M PBS solution. Primary antibodies were incubated at 4 °C overnight and secondary antibodies were incubated at room temperature for 2 h. For VGluT2 staining, guinea pig anti-VGluT2 primary antibody (Sigma-Aldrich, AB2251-I; 1:2,000) and Alexa Fluor 647 donkey anti-guinea pig secondary antibody (Jackson Laboratory, 706-605-148; 1:400) were used. For VGAT staining, mouse anti-VGAT primary antibody (Synaptic Systems, 131011; 1:1,000) and Alexa Fluor 488 donkey anti-mouse secondary antibody (Jackson Laboratory, 715-545-150; 1:400) were used. For enhancing tdTomato-labeled FN axons or climbing fibers, rabbit anti-RFP primary antibody (Rockland, 600-401-379; 1:2,000) and Alexa Fluor Cy3 donkey anti-rabbit secondary antibody (Jackson Laboratory, 711-165-152; 1:400) were used. All sections were stained with DAPI for general background labeling. We took overviews of the sections with a ×10 objective lens using a fluorescence scanner (Axio Imager 2, ZEISS) and acquired high-magnification images with a ×40 or ×63 objective lens using a confocal microscope (LSM 700, ZEISS). Images were postprocessed using ZEN (blue edition, ZEISS) and ImageJ software.

### Immunoelectron microscopy

Animals injected with AAV9-hSyn-RFP in the FN were transcardially perfused with fixative (2% PFA, 2.5% glutaraldehyde and 1% sucrose in 0.1 M PB buffer, pH 7.3). After postfixation, 100-µm brain sections were cut using a vibratome (Technical Products International). Medullary sections, including the cMAO regions, were incubated with rabbit anti-RFP primary antibody (Rockland, 600-401-379; 1:2,000) overnight at 4 °C, and subsequently in biotinylated goat anti-rabbit secondary antibody (Vector, BA-1000; 1:400) for 2 h at room temperature. RFP-labeled FN axons were visualized using avidin–biotin–peroxidase complex approach (Vector, AK-5200; volume ratio 1:1). The cMAO regions were carefully dissected and embedded in epoxy resin. Ultrathin sections were cut at 50–70 nm using an ultramicrotome (Ultracut UCT, Leica Biosystems), mounted on formvar-coated copper grids (200 mesh), and further processed for postembedding anti-GABA-immunogold labeling. The grids were rinsed in 0.05 M Tris buffer (pH 7.6) containing 0.9% saline and 0.1% Triton (TBS-Triton) and left overnight in rabbit anti-GABA primary antibody (Sigma-Aldrich, A2052; 1:1,000 in TBS-Triton) at room temperature, and further incubated for 1 h in goat anti-rabbit secondary antibody conjugated with 10-nm gold particles (Aurion, 810.311; 1:25). Ultrastructural images were captured using an electron microscope (Talos L120C TEM, Thermo Fisher Scientific) and analyzed using ImageJ.

### Single-neuron tracing

Animals were injected with AAV9-CAG-FLEX-GFP in the FN and AAVretro-CAG-Cre in the contralateral mcMAO. Mice were deeply anesthetized with an intraperitoneal injection of pentobarbital sodium solution (50 mg kg^−1^) and perfused transcardially with saline, followed by 4% paraformaldehyde (PFA) in 0.1 M phosphate buffer (PB; pH 7.4). Brains were removed immediately and postfixed overnight in 4% PFA and 0.05 M PB at 4 °C. Fixed and dehydrated brains were embedded in glycol methacrylate^39^, sliced and imaged using a previously described fMOST technique^39,66^. Propidium iodide staining (background) and GFP channels (neurite tracing) were acquired in TIFF format of 30,000 × 20,000 pixels in size. The raw data are processed into 1,000,000 data cubes of 256 × 256 × 100 voxels in size. Whole-brain images were aligned to the Allen Mouse Brain CCF^67^ using an affine transformation, nonrigid image registration method. Affine and nonrigid registration was done by Computational Morphometry Toolkit software. For single-neuron reconstruction, datasets were first semiautomatically processed using Amira software, and putative axonal boutons were recognized by the DeepBouton tool^68^. Briefly, selected images and the neighboring cubes were three-dimensionally reconstructed for tracing. Axon fibers were segmented from the extracted tree-dimensional images by thresholds. Then, the centers of axonal swellings were identified by a density-peak clustering algorithm. Nonbouton swellings were filtered out by a deep convolutional network. The putative neurite connections were automatically identified and further confirmed by two experienced operators. We included 14 single FN neurons that show detectable axonal terminals in the mcMAO regions in the final dataset for further quantification. The fraction of axons distributed in specific brain regions was normalized to the total axons of every single neuron.

### In vitro patch clamp recordings and optogenetics

Animals were deeply anesthetized with isoflurane and decapitated. Brains were quickly removed and sliced coronally using a vibratome (250 μm; Leica Biosystems, VT1000S) in an ice-cold solution containing the following: 93 mM NMDG, 93 mM HCl, 2.5 mM KCl, 1.2 mM NaHPO_4_, 30 mM NaHCO_3_, 25 mM glucose, 20 mM HEPES, 5 mM sodium ascorbate, 3 mM sodium pyruvate, 2 mM thiourea, 10 mM MgSO_4_, 0.5 mM CaCl_2_ and 5 mM N-acetyl-l-cysteine (osmolarity = 310 ± 5 mOsmol kg^−1^). Caudal medullary slices, including the cMAO regions, were collected in the same slicing medium at 35 °C for 10 min and subsequently transferred to artificial cerebrospinal fluid (ACSF, containing the following: 124 mM NaCl, 2.5 mM KCl, 1.25 mM Na_2_HPO_4_, 2 mM MgSO_4_, 2 mM CaCl_2_, 26 mM NaHCO_3_ and 20 mM d-glucose) at 35 °C for at least 30 min before recording. Slicing and ACSF solutions were bubbled constantly with 95% O_2_ and 5% CO_2_.

Whole-cell patch-clamp recordings were performed using borosilicate glass pipettes (resistance = 3–6 MΩ) filled with K^+^-based intracellular solution (124 mM potassium gluconate, 9 mM KCl, 10 mM KOH, 4 mM NaCl, 10 mM HEPES, 28.5 mM sucrose, 4 mM Na_2_ATP and 0.4 mM Na_3_GTP). All recording pipettes were supplemented with 1 mg ml^−1^ biocytin (Sigma-Aldrich) to confirm the recording locations using histology. The signal was acquired using an EPC-10 amplifier (HEKA Electronics) and digitized at 50 kHz. Optogenetic activation of ChrimsonR-expressing FN axons was performed using a pE-2 LED light source (1.6 mW; CoolLED, Andover) at 585 nm wavelength. Single pulse (10 ms), 10-Hz train (10 ms, 10% duty cycle) and 20-Hz train (10 ms, 20% duty cycle) stimulations were delivered in at least ten trials with 10-s intertrial intervals. For pharmacological experiments, AMPA receptor antagonist NBQX (10 μM, Tocris) or GABA_A_ receptor antagonist picrotoxin (100 μM, Sigma-Aldrich) was added into the ACSF bath during recordings.

### In vivo single-unit recording and optogenetics

We recorded vermal PCs at a depth of 0.5–2.0 mm and FN neurons at a depth of 2.0–2.8 mm, as measured from the cerebellar surface. Multichannel recordings (Cambridge NeuroTech, 64-channels ASSY 77H-H2) were amplified and digitized in an Intan RHD2000 Evaluation System (Intan Technology) at a 20-kHz sampling rate and were further analyzed offline using custom-written MATLAB codes. In all electrophysiological experiments combined with optogenetics, at least 40 trials were delivered with a randomized intertrial interval of 10–15 s. An Orange LED light (Thorlabs, M595F2) was used to activate ChrimsonR, a green LED light (Thorlabs, M530F2) was used to activate eOPN3 and a blue LED light (Thorlabs, M470F3) was used to activate stGtACR1, stGtACR2 and ChR2 via a patch cable (core *Φ* = 200 µm; 0.22 NA, Thorlabs). Light intensity and duration were controlled by a high-power light driver (Thorlabs, DC2100). Detailed stimulation frequency, pulse duration and duty cycle for each optogenetic protocol were stated in the corresponding figure legends.

### Behavioral experiments and optogenetics

Before the behavioral tests, animals were handled daily by experimenters and habituated to the setup for at least 1 week. For monitoring saccadic eye movements, animals were head-fixed and allowed to locomote on a cylindrical treadmill in a light- and sound-attenuated chamber. A high-speed camera (Basler, acA640-750um) equipped with a high-resolution lens (Basler, C125-0618-5M-P) was placed 4.5 cm and 59° (relative to the anteroposterior axis) in front of the left eye. An infrared LED was mounted next to the camera for illumination. Eye movements were captured at 200 fps using Basler Video Recording Software and stored for further analysis. An external trigger generated by Pulse Pal (Sanworks) was used to control camera acquisition and synchronization with the Intan RHD2000 Evaluation System (Intan Technology). The optogenetic trigger was controlled by custom-written Labview codes and recorded by Intan RHD2000 Evaluation System. All optogenetic stimulations were delivered to the left FN^E^–IO, ipsilateral to the monitored eye.

To test the effects of different optogenetic intensities, four conditions with graded intensities were given as follows: 1.15, 1.60, 2.15, and 3.00 mW (250 ms, 50 Hz and 50% duty cycle). To test the effects of different optogenetic durations, 10-ms single pulse, 50-ms single pulse, 250-ms train (50 Hz and 50% duty cycle) and 500-ms train (50 Hz and 50% duty cycle) stimulations at the intensity of 3.00 mW were given. At least 40 trials of each stimulation were given to the animals with randomized intertrial intervals between 10 s and 15 s.

In the dual optogenetic experiments (Fig. 7a–d), mcMAO inhibition (50 Hz, 50% duty cycle and 2.4 mW) started 250 ms before the 250-ms FN^E^–IO activation (50 Hz, 50% duty cycle, 3.0 mW), and coterminated with the FN^E^–IO activation. At least 40 mcMAO-inhibition trials were randomized during the FN^E^–IO-activation trials (1:4 ratio), and paired comparison was performed in the same animals.

To test the effects of FN^E^–IO terminal inhibition on spontaneous saccade, we injected an inhibitory opsin AAV1-hSyn-SIO-eOPN3-mScarlet in FN and AAVretro-CAG-Cre in the contralateral mcMAO. An optical fiber (5 mm, *Φ* = 200 μm; 0.22 NA, Thorlabs) was implanted over the mcMAO region. Mice were first habituated on the setup for at least 4 weeks to be able to perform sufficient numbers of spontaneous saccades (>3 min^−1^). We recorded the spontaneous saccade during long-term FN^E^–IO terminal inhibition (50 Hz, 50% duty cycle and 2.5 mW) for 30 min and compared those with the saccade recorded during the control condition (no optogenetics, also 30-min recording) from the same animals.

For recording body movements, animals were placed in a 15 × 11 × 20-cm^3^ arena made of transparent acrylic. A high-speed camera (Basler, acA640-750um) equipped with a high-resolution lens (Basler, C125-0618-5M-P) was placed 50 cm above the arena, and the position was fixed after verifying the focus. LED light panels were mounted around the arena for illumination. Optogenetic stimulations and camera acquisition were synchronized and reordered in the same way as described above.

### Anatomical analysis

To quantify the distribution of FN axons, raw images were processed and analyzed using custom-modified AMaSiNe pipeline^35^ with generous help from Dr. W. Choi and Dr. S.B. Paik (Korea Advanced Institute of Science and Technology). Briefly, raw images were automatically registered to the standard Allen Mouse Brain CCF^3^ (10 µm per voxel) using an affine transformation followed by a nonrigid transformation b-spline. RFP/GFP-labeled somata or axons were detected automatically by the algorithms and manually inspected to remove false-positive signals. These procedures were confirmed by at least two experimenters to ensure accuracy. To calculate the fractions of excitatory versus inhibitory FN axons in the IO, colocalization of tdTomato-labeled FN terminals and VGluT2^+^/VGAT^+^ was manually identified using ZEN software (black edition, ZEISS). Segmentation and quantification of FN^E^–IO axons in the spinal cord were analyzed using ImageJ.

### Electrophysiological analysis

All analyses for in vitro electrophysiology were performed using MATLAB software. Evoked EPSC traces were first normalized by subtracting the baseline (500 ms before optogenetics) and aligned to the photostimulation onset. For each neuron, the peak EPSC amplitudes were calculated and averaged across all trials. The same method was applied to the IPSC amplitude analysis.

For in vivo electrophysiology, raw recordings were notch-filtered at 50 Hz and band-pass filtered at 300–3,000 Hz to subtract noise and field potential signals. The SSs of PCs and FN neuron activities were sorted using JRCLUST^69^, and all spike time was stored for further analysis. CSs of PCs were sorted using an in-house developed code SpikeTrain (Neurasmus) in MATLAB^32^. We extracted CS events with amplitudes that exceed a threshold of 3× SDs of the baseline noise. We performed additional manual waveform sorting on CSs according to their distinct features of an initial spike followed by high-frequency spikelets. All in vivo electrophysiological analyses, including trigger detection, modulation identification, timing and amplitude calculations were performed using customed MATLAB codes as detailly described in our previous work^32^. Briefly, peristimulus time histograms (PSTHs) of well-isolated single units were constructed by superimposing the trigger-aligned spike time in a 5-ms bin window and presented in frequency. The baseline firing rate was calculated as the mean frequency in a 500-ms window before the trigger onset. Following the trigger, neurons with firing rate changes larger than 3× SDs of the baseline frequency were classified as modulating neurons.

### Behavioral analysis

The movement tracking in head-fix and freely moving animals was performed using a machine learning algorithm DeepLabCut^70^. To train the network, at least 300 frames from six different animals were manually labeled. For eye movement tracking, we labeled the following three positions: pupil center, medial and lateral commissures. For body movement tracking, the following four positions were manually labeled (see example in Fig. 6j): head, neck, chest and hip. Each network was trained for at least 800,000 iterations. The extracted pixel coordinates were analyzed and plotted using custom-written MATLAB codes.

For saccadic eye movement, pixel coordinates in two-dimensional video plane (672 × 512 pixels) were converted to angular eye positions using a model-based calibration approach^71^. Pupil coordinates were extracted and aligned to the mediolateral commissure axis—the horizontal eye axis was defined along the line connecting mediolateral commissures, and the vertical eye axis was orthogonal to this line. As the minimal vertical eye movement was detected in our work (Fig. 6b) and in other mouse studies^72,73^, we focused on analyzing the horizontal saccade. In the optogenetic experiments, all trials were aligned to the stimulation onsets, and we removed trials with noisy baseline (eye blink trials) by performing an iterative Grubbs’ outlier detection test (α = 0.05) on the standard deviations of baseline. The following criteria for evoked saccadic eye movements were applied based on the previous studies^72,73^: (1) baseline velocity was calculated as the average eye movement velocity within a 250-ms window before photoactivation; (2) given a 100-ms poststimulation window, we detected the first time point in which the velocity changes increased at least 3× SDs of the baseline and defined it as the saccade onset if this value was larger than 50° s^−1^; (3) trials were discarded if the eye did not move in the same direction for at least four consecutive frames (20 ms) or their peak velocities (within 150 ms after stimulation) were under 100° s^−1^. For detecting spontaneous saccades (Fig. 6e–i), we first detected eye movements presenting high velocities (>50° s^−1^) and then applied the same criteria to refine the saccadic events.

For analyzing photo-evoked body movement, trigger-aligned velocities of all labeled positions were calculated to determine whether the photoactivation elicited movements. Trials were discarded when animals presented rearing, grooming or other behaviors in which labeled positions were not tracked. In the final dataset, at least 30 trials for each condition were validated for further analysis. To achieve reliable detection in freely moving animals, we only considered movements with prominent velocity change following the optogenetics as evoked events—velocity change exceeded 6× SDs of the baseline for at least three consecutive frames (15 ms) in a 150-ms window after photoactivation onset. Baseline was calculated as the average velocity within 250 ms before photoactivation. The turning angle was defined as the angular change of the neck-head vector relative to the angle at the last frame before stimulation. The peak turning angle was calculated as the maximum value within 250 ms after stimulation.

### Statistics and reproducibility

All statistics were performed using MATLAB and GraphPad Prism. Data distribution was assumed to be normal, but this was not formally tested. Group average of neuron activity and behavioral traces were plotted as mean ± s.e.m., and sample sizes were displayed in corresponding figures. Statistical comparisons were performed by using *t*-test, ANOVA and Wilcoxon signed rank test depending on the experiment and data specificity, unless stated otherwise. Statistical significance was defined as *P* < 0.05, and annotations were *P < 0.05, **P < 0.01 and ***P < 0.001, respectively; no significant difference was denoted as NS Sample sizes were not predetermined by statistical method, but we repeated each experiment in at least three animals based on previous studies^27,74^ and experience in our laboratory. No randomization was used in our experiments as no selection bias was introduced. Data collection and analysis were not performed blind to the conditions of the experiments. No animals or data points were excluded.

### Reporting summary

Further information on research design is available in the Nature Portfolio Reporting Summary linked to this article.

## Online content

Any methods, additional references, Nature Portfolio reporting summaries, source data, extended data, supplementary information, acknowledgements, peer review information; details of author contributions and competing interests; and statements of data and code availability are available at 10.1038/s41593-023-01387-4.

## Supplementary information

Supplementary Information Supplementary Figs. 1–11, Supplementary Table 1 and Supplementary References.

Reporting Summary

Supplementary Video 1 Example evoked saccade by activating FN^E^–IO neurons. The video was recorded at 200 fps and played at 30 fps, normal (1×) and slow speed (0.125×). Stimulation protocol: 250 ms, 50 Hz, 50% duty cycle, 3 mW. N and T denote nasal and temporal direction, respectively.

Supplementary Video 2 Example evoked upper body movement by activating FN^E^–IO neurons in a freely moving mouse. The video was recorded at 200 fps and played at 30 fps, normal (1×) and slow speed (0.125×). Stimulation protocol: 250 ms, 50 Hz, 50% duty cycle, 3 mW.

Supplementary Data Supporting data for Supplementary Figs. 1, 2, 5, 7, 8 and 10.

## Data Availability

Source data used to make each of the figures are provided with this paper. Raw data are available from the corresponding author (Z.G.) upon reasonable request. Source data are provided with this paper.

## Source data

Source Data Fig. 1 Statistical source data.
Source Data Fig. 2 Statistical source data.
Source Data Fig. 3 Statistical source data.
Source Data Fig. 4 Statistical source data.
Source Data Fig. 5 Statistical source data.
Source Data Fig. 6 Statistical source data.
Source Data Fig. 7 Statistical source data.
Source Data Extended Data Fig. 1 Statistical source data.
Source Data Extended Data Fig. 2 Statistical source data.
Source Data Extended Data Fig. 3 Statistical source data.
Source Data Extended Data Fig. 4 Statistical source data.
Source Data Extended Data Fig. 5 Statistical source data.
Source Data Extended Data Fig. 6 Statistical source data.
Source Data Extended Data Fig. 7 Statistical source data.
Source Data Extended Data Fig. 9 Statistical source data.
Source Data Extended Data Fig. 10 Statistical source data.

## Code availability

All custom analysis codes generated in MATLAB can be found in the following repository: https://github.com/XiaoluOne/Excitatory-FN-IO-paper.git. The data acquisition codes created in Labview, and other custom codes in MATLAB are available from the corresponding author (Z.G.) upon reasonable request.
