# Supplementary material for: Excitatory nucleo-olivary pathway shapes cerebellar outputs for motor control
Source: Nat Neurosci. 2023 Jul 20;26(8):1394–406. doi: 10.1038/s41593-023-01387-4 (PMC10400430; doi:10.1038/s41593-023-01387-4)
Supplement: Supplementary file 1 — Supplementary Figs. 1–11, Supplementary Table 1 and Supplementary References. [file 41593_2023_1387_MOESM1_ESM.pdf]

nature neuroscience

Article https://doi.org/10.1038/s41593-023-01387-4

Excitatory nucleo-olivary pathway shapes cerebellar outputs for motor control

In the format provided by the authors and unedited

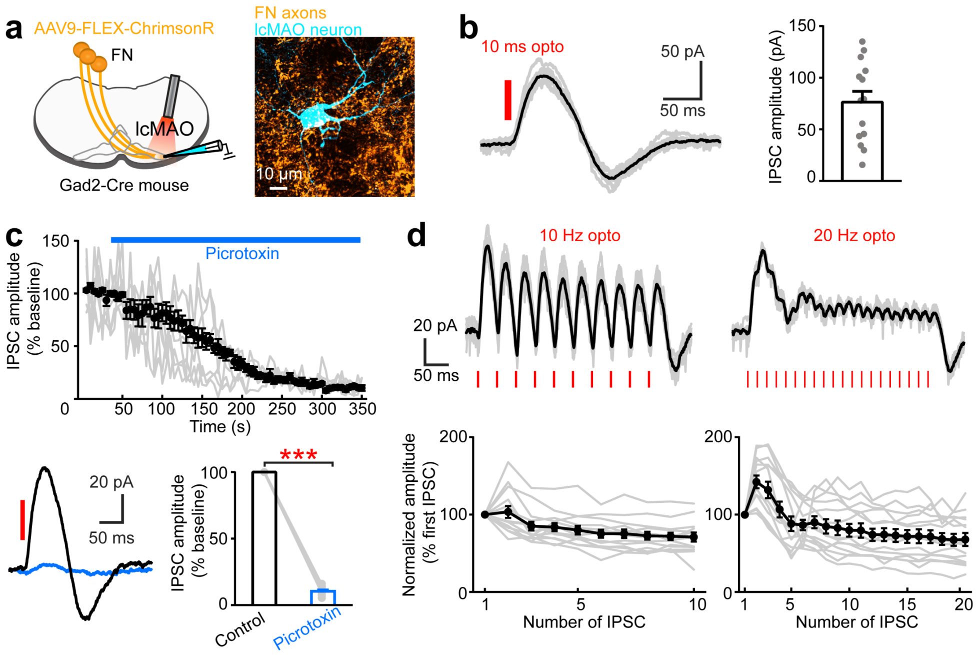

Supplementary Fig.1 | Activation of FN terminals evokes IPSCs in IcMAO neurons. a, Left: schematic illustrating *in-vitro* whole-cell recording of IcMAO neurons while photoactivating FN axons. Right: an example IcMAO neuron (biotin labeled, 1/14 cells displayed) embedded with dense FN axons. **b**, Left: photo-evoked IPSC traces from an example IcMAO neuron (dark: average trace, gray: 10 individual sweeps). Right: IPSC amplitudes of 14 cells from 5 mice (mean + s.e.m.). **c**, Upper: reduction of IPSC amplitudes over time following picrotoxin infusion. Black: group average, gray: individual cells (n = 6 from 4 mice, mean ± s.e.m.). Lower left: IPSC traces of an example cell before (black) and after (blue) picrotoxin infusion. Lower right: comparison of IPSC amplitudes before and after picrotoxin infusion (n = 6 cells from 4 mice, paired t-test, $P = 3.73 \times 10^{-9}$). Gray dots represent individual cells, bars represent mean + s.e.m.. Amplitudes are normalized to control. **d**, Upper: evoked IPSC traces following 10-Hz and 20-Hz train stimulations from an example cell. Lower: IPSC amplitudes flowing optogenetic pulses (dark: average amplitudes, gray: amplitudes of individual cells, mean ± s.e.m., n = 14 from 5 mice). Amplitudes are normalized to the first IPSC amplitude.

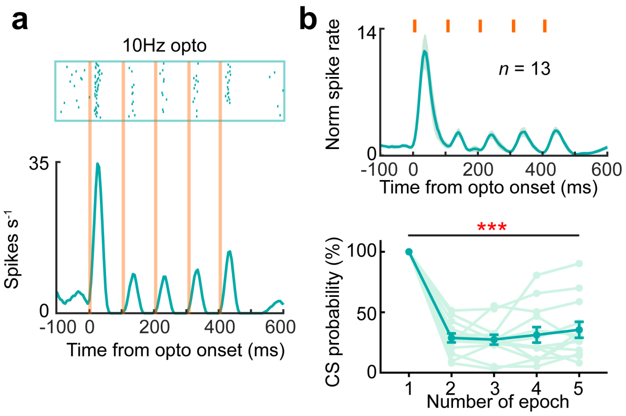

Supplementary Fig.2 | 10-Hz train photoactivations of the FN axons in the mcMAO evoke CSs. Related to (**Fig. 3**). **a**, Raster plots and PSTH traces of CS activity from an example vermal PC, following 10-Hz train stimulation of FN^{E}-IO axons (10-ms photoactivation, 10% duty cycle, 5 pulses per train). **b**, Average CS activity trace (upper) and evoked CS probabilities (lower) in response to 10-Hz train photoactivation, indicating a higher CS probability following the first pulse and reduced probabilities for the subsequent pulses (*n* = 13 cells from 3 mice, one-way ANOVA with Tukey's multiple comparisons, ***$P = 2.27\times10^{-11}$). Evoked CS probabilities are normalized to the first response. Upper: PSTH is plotted as mean ± s.e.m.; lower: lighter dots and bars represent individual neurons, darker dots and bars represent mean ± s.e.m..

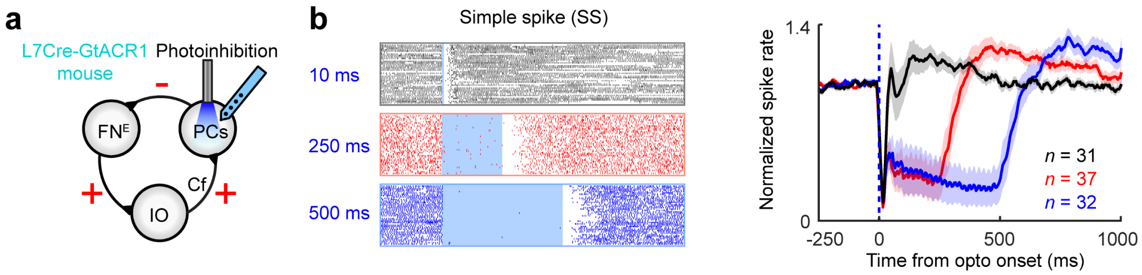

Supplementary Fig.3 | Simple spike (SS) activity following single-pulse and train photoinhibition of vermal PCs. Related to (**Fig. 4a-d**). **a**, Schematic illustrating vermal PC recording while photoinhibiting PCs in the same region, same as (**Fig. 4a**). **b**, Left: raster plots of SSs from an example PC, following 10-ms single pulse (black), 250-ms train (red, 50 Hz, 50% duty cycle), and 500-ms train (blue, 50 Hz, 50% duty cycle) photoinhibition protocols. The optogenetic light intensity was 3.0 mW for all protocols. Right: average SS activity from all PCs (mean ± s.e.m.), normalized to the baseline SS spike rates. See cell number under each condition in the panel, *n* = 4 mice. The dashed line indicates the onset of photoinhibition.

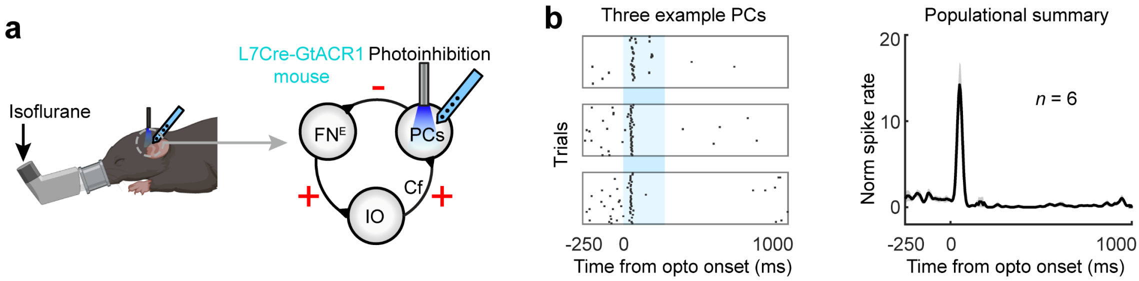

Supplementary Fig.4 | PC inhibition and recording in anesthetized animals. a, Experiment schematics: recordings were performed on anesthetized mice (1% isoflurane inhalation) in which photoinhibition of PCs did not induce movements. **b,** Left: raster plots of CS activity from three example PC recordings. Right: PC inhibition triggered well-timed CSs in anesthetized mice. Summary of PSTH is plotted as mean ± s.e.m. (*n* = 6 PCs from 3 mice).

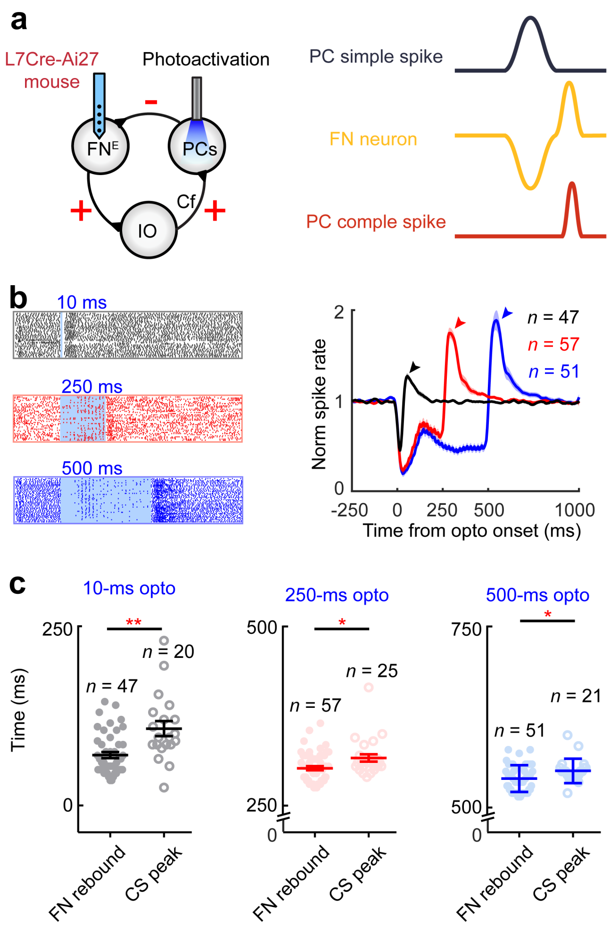

Supplementary Fig.5 | Activating vermal PCs induces post-inhibitory rebound firing in FN neurons. Related to (**Fig. 4e-h**). **a**, Left: schematic of FN recording while photoactivating vermal PCs. Right: hypothetical effects of SS activation on the suppression of FN neuron activity and subsequent post-inhibitory rebound activation, which can drive CSs via the FN^E-IO module. **b**, Left: raster plots of an example FN neuron following PC stimulations by a 10-ms single pulse (black), 250-ms train (50 Hz, 50% duty cycle), and 500-ms train (50 Hz, 50% duty cycle). Right: average PSTHs of all FN neurons (mean ± s.e.m., see numbers of recordings in the panel). The photoactivation intensity was 3.0 mW for all protocols. Arrowheads denote the post-inhibitory rebound activation. **c**, Comparison of the peak timings between FN post-inhibitory rebound and photo-evoked CSs (see experiments in **Fig. 4e-h**) for three stimulation conditions, showing that CSs follow FN rebound activation (two-sided unpaired *t*-test with Welch's correction, see numbers of recordings in the panel, left to right: $P = 0.0031$, 0.019, and 0.024). Dots and circles represent individual neurons, bars represent mean ± s.e.m..

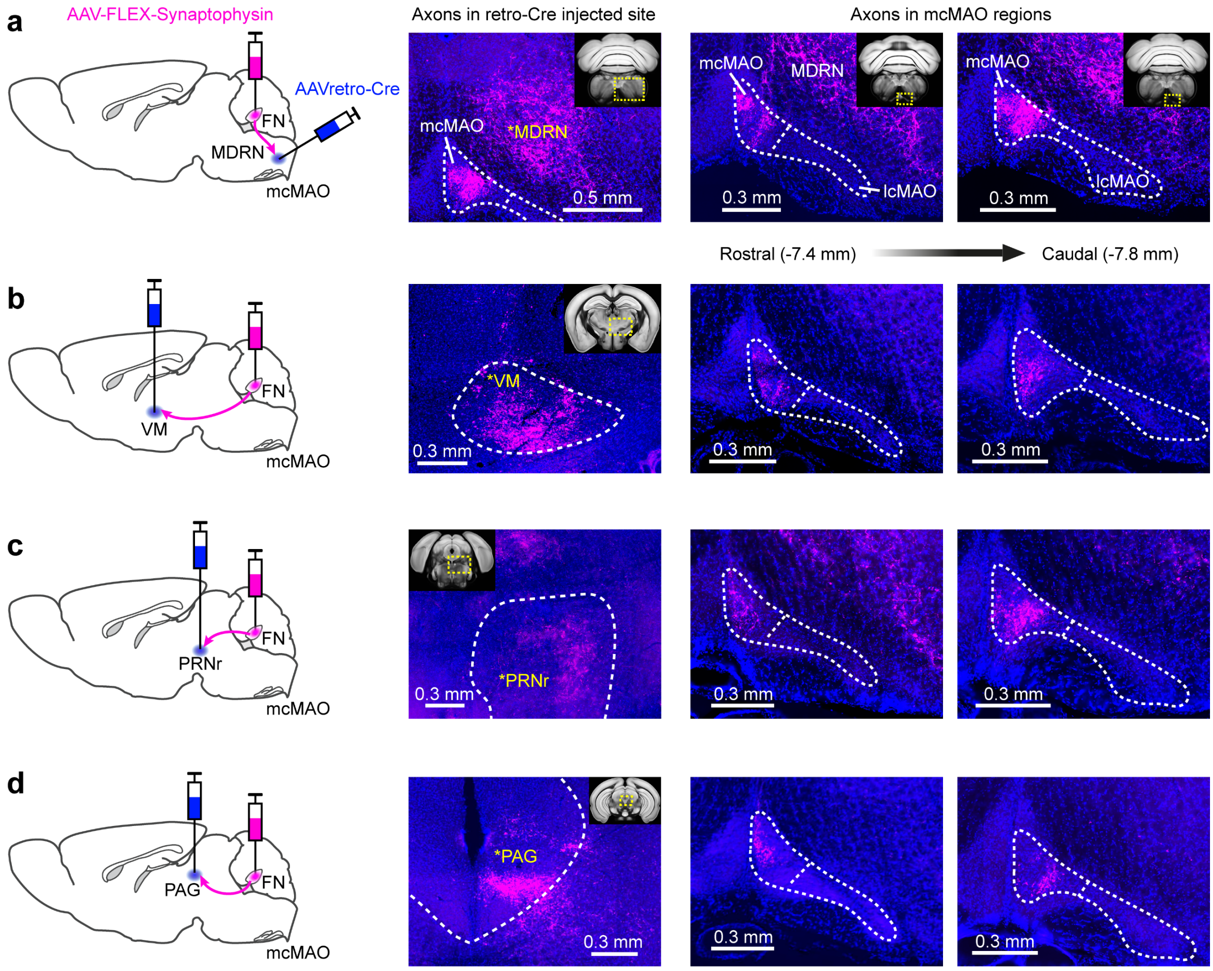

Supplementary Fig.6 | Targeted tracing of distinct FN subpopulations confirms that a wide range of FN output neurons collateralize to the mcMAO. a, Left: schematic showing injections of AAV-FLEX-Synaptophysin in the FN and AAVretro-Cre in the MDRN to specifically label MDRN-projecting FN neurons. Middle: representative image confirming the FN projection in the MDRN (yellow asterisk). Right: representative images showing that MDRN-projecting FN neurons also project to the mcMAO. 1/3 mice displayed. **b-d**, Same as **a**, but for labeling the FN subpopulations that project to the VM (1/3 mice displayed), PRNr (1/2 mice displayed), and PAG (1/3 mice displayed). All these FN subpopulations send axon collaterals to the mcMAO. MDRN: medullary reticular nucleus, VM: ventral medial nucleus of the thalamus, PRNr: pontine reticular nucleus, PAG: periaqueductal gray.

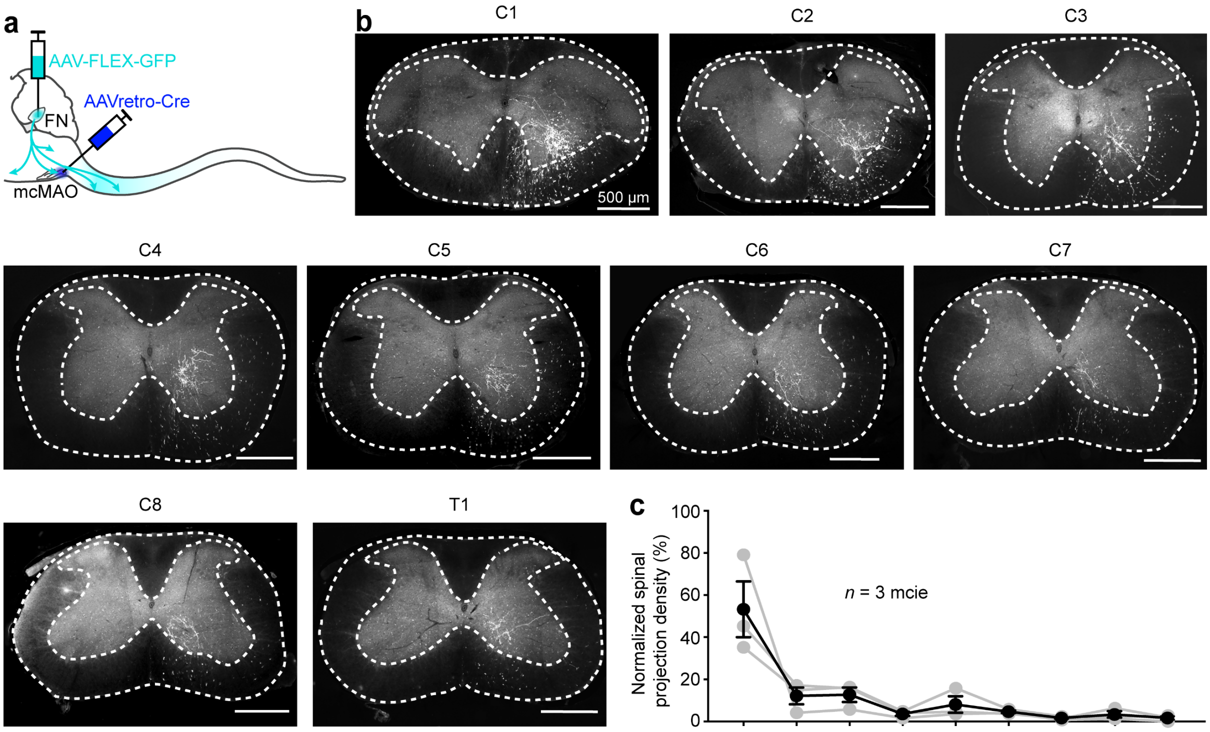

Supplementary Fig.7 | FN^{E}-IO neurons project to the contralateral spinal cord. Related to (**Fig. 5a-c**). **a**, Tracing strategy for labeling FN^{E}-IO neurons, same as (**Fig. 5a**). **b,** Representative images of spinal cord, showing axonal labeling in the contralateral ventral horn of the cervical and thoracic sections (C1 to T1, 1/3 mice displayed). **c**, Summary of the projection density across different levels. Projection density was normalized to the total spinal labeling of each mouse (*n* = 3 mice). Gray dots: individual mice, black dots and bars: mean ± s.e.m..

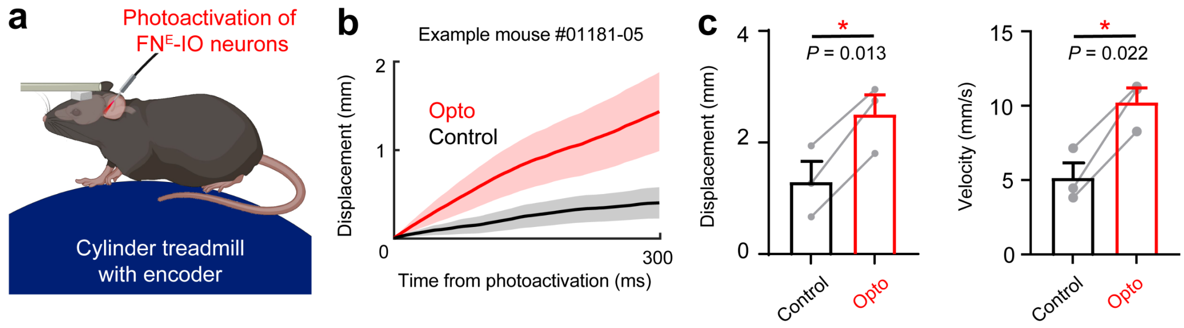

Supplementary Fig.8 | Photoactivation of FN^E-IO evokes attempted body movements in head-fixed mice. a, Experiment schematics. Same as **(Fig. 6a),** animals were head-fixed on a freely rotating cylinder treadmill. Attempted body movements were recorded by the movements of the treadmill. **b,** Treadmill displacement of an example mouse reflecting attempted body movement during photoactivation (250-ms duration, 50% duty cycle, 3 mW). Control: sham optogenetic activation. Traces are plotted as mean ± s.e.m. (n = 23 optogenetic trials, n = 22 control trials). **c,** Summary of treadmill displacement and velocity from 3 mice (two-sided paired t-test, see the exact P values in the panel). Dots represent individual mice and bars represent mean + s.e.m..

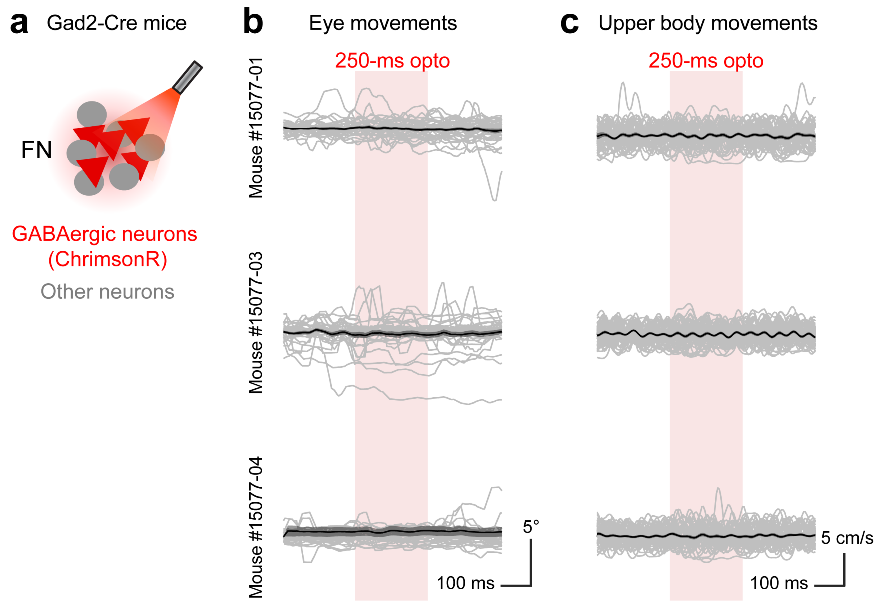

Supplementary Fig.9 | Activating GABAergic FN neurons does not trigger movements. a, Experiment schematics. AAV9-FLEX-ChrimsonR was injected in the FN of Gad2-Cre mice (*n* = 3). **b**, Eye positions of three mice during 250-ms photoactivation (3 mW, 250-ms duration, 50% duty cycle). Gray traces represent individual trials, black traces represent mean ± s.e.m. (upper to lower: *n* = 40, 36, and 38 trials). **c**, Same as **(b)**, but for the velocity of upper body movements of the same animals. Gray traces represent individual trials, black traces represent mean ± s.e.m. (upper to lower: *n* = 48, 50, and 48 trials).

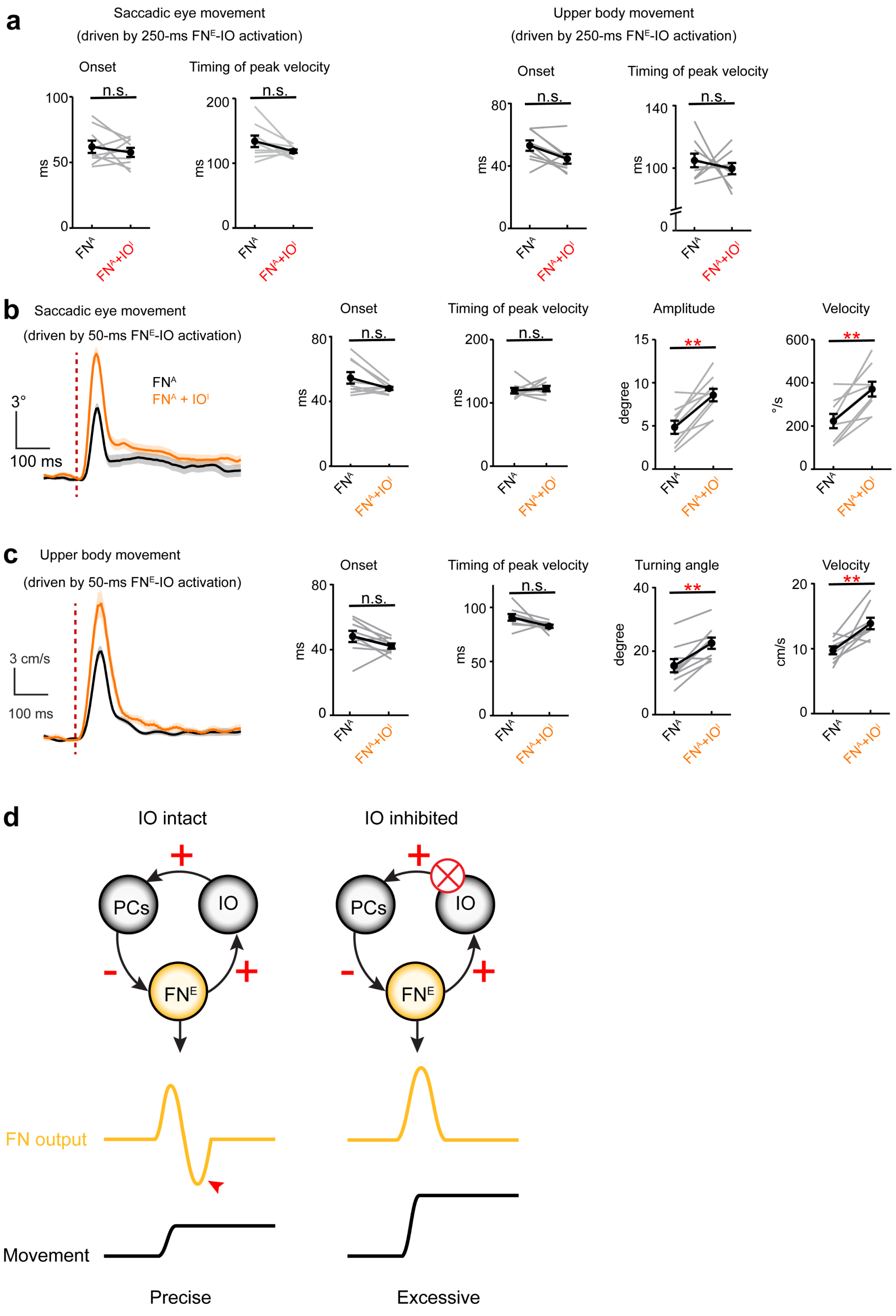

Supplementary Fig.10 | Feedback CSs driven by the FN^E-IO pathway modulates precise saccadic and upper body movements. Related to (**Fig. 7**). **a**, Left two panels**:** the onset timing and peak velocity timing of FN^E-IO-driven (FN^A, 250-ms photoactivation) saccades, with and without photoinhibition of the mcMAO neurons (IO^I). Right two panels: same as the left but for the FN^E-IO-driven upper body movements. Inhibiting mcMAO had no effect on the movement onset timing or the timing of peak velocity (two-sided Wilcoxon matched-pairs signed rank test, *n* = 9 mice, left to right: *P* = 0.50, 0.15, 0.055, and 0.65). Black dots and bars represent mean ± s.e.m.. **b**, Left: inhibiting the mcMAO enhances the amplitude of the FN^E-IO driven saccade (50-ms photoactivation) in an example

mouse. Traces are plotted as session average (mean ± s.e.m.). Right: comparisons of the saccade onset, timing of peak velocity, amplitude, and maximum velocity with and without photoinhibition of the mcMAO (two-sided Wilcoxon matched-pairs signed rank test, n = 9 mice, left to right: P = 0.074, 0.73, 0.0078, and 0.0078). Black dots and bars represent mean ± s.e.m.. **c**, Same as **b**, but for the comparison of the FN^E-IO driven (50-ms photoactivation) upper body movement with and without photoinhibition of the mcMAO. Traces are plotted as session average (mean ± s.e.m.). Left to right: two-sided Wilcoxon matched-pairs signed rank test, n = 9 mice, P = 0.13, 0.074, 0.0078, and 0.0078. Black dots and bars represent mean ± s.e.m.. **d**, Illustrative summary depicting the contribution of the excitatory nucleo-olivary pathway to motor control. Activation of FN^E-IO neurons results in feedback inhibition of the medial cerebellar output via the excitatory FN^E-IO-PC loop. This feedback loop constrains the duration and amplitude of FN output, thereby precisely controlling the amplitude and velocity of movement. The mcMAO inhibition abolishes the post-faciliatory feedback inhibition, resulting in excessively strong FN output and hence movement overshoot.

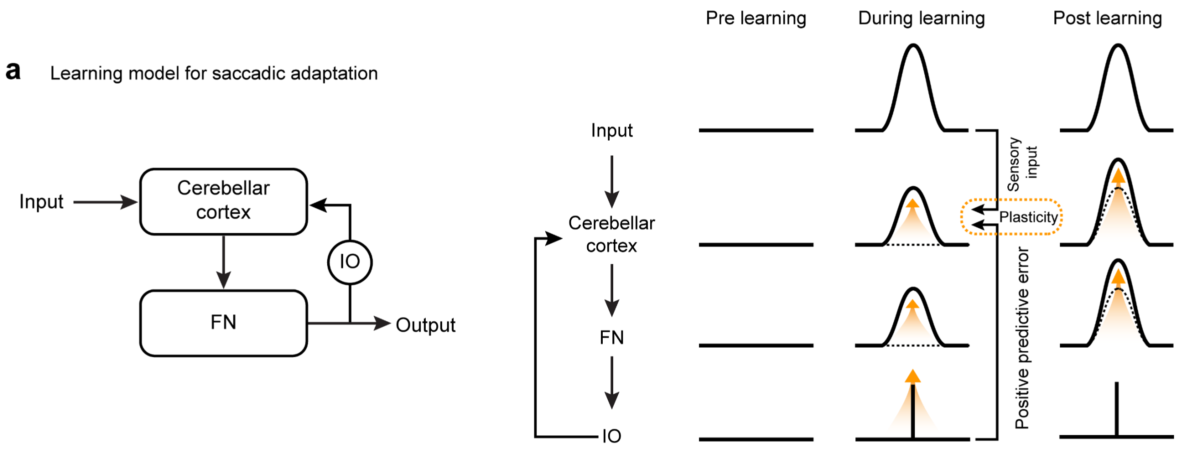

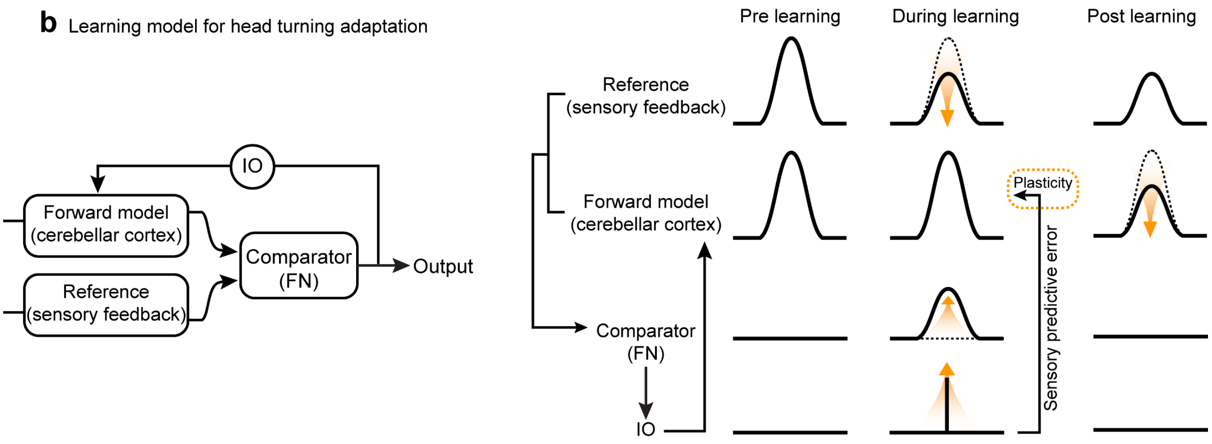

Supplementary Fig.11 | Hypothetical learning models for saccadic adaptation and head turning adaptation using the FN^E-IO modules. We propose two hypothetical implementations of the FN^E-IO modules for motor learning, inspired by experimental evidence from two studies. **a**, Learning model and signaling cascade for the emergence of CSs during saccadic adaptation[1]. During learning, novel synaptic inputs to the cerebellar cortex may activate specific groups of cortical neurons. Here we illustrate the abstract weight of cortical activity, not the excitatory or inhibitory nature of particular synapses. Cortical outputs activate the FN^E-IO neurons, which provide CS feedback to inform the cerebellar cortex about this unexpected motor command as a positive predictive error. Repetitive activation of the same FN^E-IO neurons will induce plasticity at the cortical layer and reinforce the output of these FN^E-IO neurons. The FN^E-IO-cortical loop ensures the maintenance of acquired CS responses, as well as the saccadic adaptation (post learning). **b**, Learning model and signaling cascade for head turning adaptation[2]. We consider the FN^E-IO neurons as a comparator in which the vestibular reference (sensory feedback signal) and the predictive motor command (cerebellar forward model) are compared during head turning. Coherent sensory feedback and predictive motor command result in baseline FN^E-IO activity before learning. A torque is applied to the head during head turning adaptation. The difference between the expected movement position and sensory feedback of the actual head position generates a sensory prediction error signal in the FN^E-IO neurons and hence CSs in the cerebellar cortex. The sensory predictive error carried by CS updates the cerebellar forward model, resulting in a reduction of FN activity after adaptation (post learning).

Supplementary Table.1 | Stereotaxic coordinates of nuclei for injection and optical fiber implantation.

Nuclei	AP (mm)	ML (mm)	Depth (mm)
Fastigial nucleus*	-2.7	0.8	2.4
Medial cMAO (mcMAO)*	-3.1	0.1	5.1
Lateral cMAO (lcMAO)*	-3.0	0.4	5.3
Medullary reticular nucleus*	-3.4	0.6	4.7
Pontine reticular nucleus*	-1.0	1.0	3.6
Periaqueductal gray*	-0.6	0.2	2.7
Ventromedial thalamic nucleus	-1.4	0.8	4.2

*The starting location is defined as the anterior tip of the interparietal bone, otherwise as bregma. Viral injection volume was 20-50 nL. AP: anterior posterior, ML: medial lateral.

Supplementary references

1. Catz, N., Dicke, P.W. & Thier, P. Cerebellar complex spike firing is suitable to induce as well as to stabilize motor learning. *Curr Biol* **15**, 2179-2189 (2005).
2. Brooks, J.X., Carriot, J. & Cullen, K.E. Learning to expect the unexpected: rapid updating in primate cerebellum during voluntary self-motion. *Nature neuroscience* **18**, 1310-1317 (2015).
