# Supplementary material for: Excitatory nucleo-olivary pathway shapes cerebellar outputs for motor control
Source: Nat Neurosci. 2023 Jul 20;26(8):1394–406. doi: 10.1038/s41593-023-01387-4 (PMC10400430; doi:10.1038/s41593-023-01387-4)
Supplement: Supplementary file 2 — Reporting Summary [file 41593_2023_1387_MOESM2_ESM.pdf]

nature portfolio

Corresponding author(s): Zhenyu Gao

Last updated by author(s): Jun 1, 2023

Reporting Summary

Nature Portfolio wishes to improve the reproducibility of the work that we publish. This form provides structure for consistency and transparency in reporting. For further information on Nature Portfolio policies, see our Editorial Policies and the Editorial Policy Checklist.

Statistics

For all statistical analyses, confirm that the following items are present in the figure legend, table legend, main text, or Methods section.

n/a	Confirmed	
☐	☒	The exact sample size (*n*) for each experimental group/condition, given as a discrete number and unit of measurement
☐	☒	A statement on whether measurements were taken from distinct samples or whether the same sample was measured repeatedly
☐	☒	The statistical test(s) used AND whether they are one- or two-sided<br>*Only common tests should be described solely by name; describe more complex techniques in the Methods section.*
☒	☐	A description of all covariates tested
☐	☒	A description of any assumptions or corrections, such as tests of normality and adjustment for multiple comparisons
☐	☒	A full description of the statistical parameters including central tendency (e.g. means) or other basic estimates (e.g. regression coefficient) AND variation (e.g. standard deviation) or associated estimates of uncertainty (e.g. confidence intervals)
☐	☒	For null hypothesis testing, the test statistic (e.g. *F*, *t*, *r*) with confidence intervals, effect sizes, degrees of freedom and *P* value noted<br>*Give P values as exact values whenever suitable.*
☒	☐	For Bayesian analysis, information on the choice of priors and Markov chain Monte Carlo settings
☒	☐	For hierarchical and complex designs, identification of the appropriate level for tests and full reporting of outcomes
☒	☐	Estimates of effect sizes (e.g. Cohen's *d*, Pearson's *r*), indicating how they were calculated

Our web collection on statistics for biologists contains articles on many of the points above.

Software and code

Policy information about availability of computer code

Data collection	In-vitro patch clamp recording data was acquired using a HEKA amplifier (EPC-10). In-vivo electrophysiological recording data was collected using Intan Evaluation System (RHD2000, Intan Technology). Behavior data was collected using Basler camera (acA640-750um) equipped with a high-resolution lens (C125-0618-5M-P, Basler). Fluorescence microscopy images were collected using wide-field fluorescence scanner (Axio Imager 2, ZEISS) and confocal microscope (LSM 700, ZEISS). Ultrastructural images were captured using an electron microscope (Talos L120C TEM, Thermo Fisher). Single-neuron tracing data was acquired using fMOST technique in H.G. lab (Gong, et al. 2016, Nat Commun; Wang, et al. 2021, Cell reports).
Data analysis	Electrophysiological and behavior data were analyzed using Matlab 2014 and 2017. Histological images were processed and analyzed using ZEN (blue edition, ZEISS) and ImageJ. All statistics were performed using Matlab 2017 and GraphPad Prism 6.<br><br>We used machine learning algorithm DeepLabCut to track the eye and body movements (Nath, et al. 2019, Nat Protoc); custom-modifed AMaSiNe pipeline to analyze the axon and soma labeling (Song, et al. 2020, Cell reports); for single-neuron tracing, Computational Morphometry Toolkit software was used to register raw images, and DeepBouton tool was used to identified axonal boutons (Cheng, et al. 2019, Front Neuroinform).<br><br>All custom analysis codes generated in Matlab can be found in the following repository: https://github.com/XiaoluOne/Excitatory-FN-IO-paper.git. The data acquisition codes created in Labview, and other custom codes in Matlab are under development for other unpublished works, therefore only available from the corresponding author (Z.G.) upon reasonable request.

For manuscripts utilizing custom algorithms or software that are central to the research but not yet described in published literature, software must be made available to editors and reviewers. We strongly encourage code deposition in a community repository (e.g. GitHub). See the Nature Portfolio guidelines for submitting code & software for further information.

Data

Policy information about availability of data

All manuscripts must include a data availability statement. This statement should provide the following information, where applicable:

- Accession codes, unique identifiers, or web links for publicly available datasets
- A description of any restrictions on data availability
- For clinical datasets or third party data, please ensure that the statement adheres to our policy

Allen Mouse Brain CCF (https://mouse.brain-map.org/static/atlas) was used as an reference template for anatomical analysis. Source data used to make each of the figures are provided with this paper. Raw data are being used for preparing other unpublished works, therefore only available from the corresponding author (Z.G.) upon reasonable request.

Human research participants

Policy information about studies involving human research participants and Sex and Gender in Research.

Reporting on sex and gender	N/A
Population characteristics	N/A
Recruitment	N/A
Ethics oversight	N/A

Note that full information on the approval of the study protocol must also be provided in the manuscript.

Field-specific reporting

Please select the one below that is the best fit for your research. If you are not sure, read the appropriate sections before making your selection.

☒ Life sciences ☐ Behavioural & social sciences ☐ Ecological, evolutionary & environmental sciences

For a reference copy of the document with all sections, see nature.com/documents/nr-reporting-summary-flat.pdf

Life sciences study design

All studies must disclose on these points even when the disclosure is negative.

Sample size	The sample sizes for anatomical, electrophysiological and behavioral experiments were decided similar to the sample sizes used in this fields (Calame, et al. 2023, Nat Neurosci; Kim, et al. 2020, Nat Neurosci): more than 3 animals were used in each experiment, dozens to hundreds of neurons were recorded to access the electrophysiological dynamics in response to manipulations or correlation with behavior.
Data exclusions	No data was excluded.
Replication	All results were replicated at least in multiple animals (n >= 3).
Randomization	No randomization was used in our experiments as no selection bias was introduced.
Blinding	Investigators were not blinded to execute experiments or to analyze data as data acquisition and analysis were performed based on objective measurements by at least two experimenters.

Reporting for specific materials, systems and methods

We require information from authors about some types of materials, experimental systems and methods used in many studies. Here, indicate whether each material, system or method listed is relevant to your study. If you are not sure if a list item applies to your research, read the appropriate section before selecting a response.

Materials & experimental systems

n/a	Involved in the study
☐	☒ Antibodies
☒	☐ Eukaryotic cell lines
☒	☐ Palaeontology and archaeology
☐	☒ Animals and other organisms
☒	☐ Clinical data
☒	☐ Dual use research of concern

Methods

n/a	Involved in the study
☒	☐ ChIP-seq
☒	☐ Flow cytometry
☒	☐ MRI-based neuroimaging

Antibodies

Antibodies used	Guinea pig anti-VGluT2 primary antibody (1:2000, Sigma-Aldrich, polyclonal, lot No. AB2251-I);<br>Mouse anti-VGAT primary antibody (1:1000, Synaptic Systems, monoclonal, lot No. 131011);<br>Rabbit anti-RFP primary antibody (1:2000, Rockland, polyclonal, lot No. 600-401-379);<br>Rabbit anti-GABA primary antibody (1:1000 in TBS-Triton, Sigma, polyclonal, lot No. A2052);<br>Alexa fluor® 647 donkey anti-guinea pig secondary antibody (1:400, Jackson, polyclonal, lot No. 706-605-148);<br>Alexa fluor® 488 donkey anti-mouse secondary antibody (1:400, Jackson, polyclonal, lot No. 715-545-150);<br>Alexa fluor® Cy3 donkey anti-rabbit secondary antibody (1:400, Jackson, polyclonal, lot No. 711-165-152);<br>biotinylated goat anti-rabbit secondary antibody (1:400, Vector, polyclonal, lot No. BA-1000);<br>goat anti-rabbit secondary antibody conjugated with 10-nm gold particles (1:25, Aurion, monoclonal, lot No. 810.311).
Validation	Here are the references for antibody validation on mouse brain slices:<br>Guinea pig anti-VGluT2 primary antibody: Neuron. 2018 Apr 18;98(2):306-319.e7. doi: 10.1016/j.neuron.2018.03.010.<br>Mouse anti-VGAT primary antibody: Nature Neuroscience. 2022 May;25(5):617-629. doi: 10.1038/s41593-022-01067-9.<br>Rabbit anti-RFP primary antibody: Cell Reports. 2022 Jun 14;39(11):110953. doi: 10.1016/j.celrep.2022.110953.<br>Rabbit anti-GABA primary antibody: Cerebral Cortex. 2010 May;20(5):1092-108. doi: 10.1093/cercor/bhp181.<br>Alexa fluor® 647 donkey anti-guinea pig secondary antibody: Nature Neuroscience. 2023 Mar;26(3):416-429. doi: 10.1038/s41593-022-01240-0. Epub 2023 Jan 12.<br>Alexa fluor® 488 donkey anti-mouse secondary antibody: BMC Biol. 2023 Apr 26;21(1):96. doi: 10.1186/s12915-023-01604-3.<br>Alexa fluor® Cy3 donkey anti-rabbit secondary antibody: Nat Commun. 2023 May 22;14(1):2939. doi: 10.1038/s41467-023-38583-6.<br>biotinylated goat anti-rabbit secondary antibody: Elife. 2023 May 23;12:e87495. doi: 10.7554/eLife.87495.<br>goat anti-rabbit secondary antibody conjugated with 10-nm gold particles: see validation image at: https://www.generon.co.uk/other-products-186/donkey-anti-rabbit-igg-h-l-10nm-339000238.html

Animals and other research organisms

Policy information about studies involving animals; ARRIVE guidelines recommended for reporting animal research, and Sex and Gender in Research

Laboratory animals	8-16 weeks old Wild-type C57BL/6J (000664), transgenic VGluT2-ires-Cre (016963), Gad2-ires-Cre (010802), L7-Cre (004146), Ai27D (012567), and R26-LNL-GtACR1-Fred-Kv2.1 (033089) mice were obtained from Jackson Laboratory; GlyT2-ires-Cre mice were originally obtained from Prof. S. Dieudonné (Institut de Biologie de l'ENS) by courtesy. We crossed the L7-Cre mice with the Ai27D mice to express excitatory ChR2 in PCs (L7Cre-Ai27), and crossed the L7-Cre mice with the R26-LNL-GtACR1-Fred-Kv2.1 mice to express inhibitory GtACR1 specifically in PCs (L7Cre-GtACR1).
Wild animals	This study did not involve wild animals.
Reporting on sex	Both male and female animals were used in this study.
Field-collected samples	This study did not involve samples collected from the field.
Ethics oversight	All animal experiments in this study were approved by the institutional animal welfare committee of Erasmus MC in accordance with Central Authority for Scientific Procedures on Animals guidelines.

Note that full information on the approval of the study protocol must also be provided in the manuscript.
